# Dispersal corridors enhance modelled near-bed larval connectivity across deep-sea protected area networks

**DOI:** 10.1038/s41559-026-03172-5

**Published:** 2026-10-23

**Authors:** Molly Kendall James, Amelia Elizabeth Henrietta Bridges, Daniel O. B. Jones, Charlotte Hannah Clubley, Antony Mark Knights, Danielle S. W. DeJonge, Kerry Louise Howell

**Affiliations:** 1 Plymouth Marine Laboratory, Plymouth, UK; 2 School of Marine and Biological Sciences, University of Plymouth, Plymouth, UK; 3 National Oceanography Centre, Southampton, UK; 4 Department of Ecoscience, Aarhus University, Aarhus, Denmark; 5 School of Biological, Earth and Environmental Sciences, University College Cork, Cork, Republic of Ireland; 6 Joint Nature Conservation Committee, Aberdeen, UK; 7 Present Address: Sharjah Marine Science Research Centre, University of Khorfakkan, Khor Fakkan, United Arab Emirates; 8 Present Address: Plymouth Marine Laboratory, Plymouth, UK

**Keywords:** Ecological modelling, Ocean sciences

## Abstract

The Clarion–Clipperton Zone (CCZ) is the focal region for imminent deep-sea nodule mining, with over 1.1 million square kilometres already under exploration licence. Although the International Seabed Authority has designated Areas of Particular Environmental Interest (APEIs) to protect regional biodiversity from mining impacts, there has been no formal assessment of whether this network functions as a coherent connected system. Here we use Lagrangian dispersal modelling and network analysis to provide a region-wide assessment of modelled larval connectivity across the CCZ and show that simulated larval exchange among APEIs is extremely limited. Under physically driven near-bed dispersal and reasonable pelagic larval dispersal times of 19–69 days, fewer than 0.02% of particles reach another APEI, with inter-APEI gaps exceeding modelled dispersal capacity. CCZ-wide dispersal is structured into four semi-isolated connectivity communities, and under mining disturbance when self-supply is removed from Areas of Mining Interest, unprotected non-mining areas emerge as the dominant modelled source of replenishment. Strategic corridor additions increase inter-APEI dispersal 406-fold and nearly doubles modelled replenishment from protected areas under disturbance, providing a reproducible, connectivity-informed evidence base for conservation design ahead of commercial mining.

## Abstract (web summary)

Using Lagrangian larval dispersal modelling and network analysis, the authors show that dispersal is extremely limited among Areas of Particular Environmental Interest within the deep-sea Clarion–Clipperton Zone. However, dispersal can be enhanced by the strategic addition of corridors.

## Main

The deep ocean is rapidly becoming the next frontier for commercial mineral extraction. At the heart of this is the Clarion–Clipperton Zone (CCZ), a 6 million-km^2^ swathe of the central Pacific seabed of interest for mining polymetallic nodules—a type of rock rich in minerals critical to modern technologies^1^. With over a million square kilometres already under licence for exploration^2^, the developments for mining the deep sea are accelerating, outpacing our understanding of its ecosystems^3,4^. The CCZ is not only species-rich but also ecologically complex^5^, and managing its biodiversity in the face of industrial disturbance remains a formidable challenge^6^.

To help protect regional biodiversity from the potential effects of mining, the International Seabed Authority (ISA)^7^ established a system of protected areas known as Areas of Particular Environmental Interest (APEIs). These were designed to represent diverse habitats and buffer mining impacts, but connectivity was not a formal design criterion^8–10^. A further expansion in 2021 brought the total protected area to nearly 2 million km^2^ (ref. ^11^), with an increased prominence of connectivity in ISA policy^12^.

Connectivity is critical to marine spatial planning^13^, central to conservation targets^14^ and important in maintaining ecological attributes^15^, such as biodiversity^16^. In the deep sea, many species depend on larval dispersal to link fragmented populations^17^. Ocean currents carry larvae between habitats, helping to preserve genetic diversity and enabling recolonization after disturbances such as mining events^18,19^. Limited connectivity may represent a constraint on the capacity of isolated populations to recover from disturbance—a concern relevant to mining scenarios. Consequently, a key component of an effective and self-sustaining network of Marine Protected Areas (MPAs)/APEIs is coherence^20,21^; however, the spatial arrangement of the APEIs, and thus their capacity to function as a connected network, has not previously been assessed at the region-wide scale.

Assessing APEI network coherence remains challenging owing to the data scarcity typical of abyssal environments, including sparse biological records, limited near-bed oceanographic observations and near-absent empirical estimates of larval dispersal for CCZ taxa^8^. This has left a critical gap in spatial planning at a moment when mining developments are accelerating and ISA frameworks increasingly require connectivity evidence to inform area-based management^12,22^. Here, we apply larval dispersal modelling and network analysis to estimate circulation-permitted larval exchange across the CCZ, providing the first region-wide assessment of APEI network coherence, using GOFS 3.1 HYCOM as the best-available reanalysis product for regional-scale connectivity where near-bed observational data remain extremely sparse. To address biological data scarcity, we adopt an ‘everything is everywhere’ approach^23^, assuming uniform larval release potential across the sea floor, which reveals all physically plausible dispersal pathways without embedding uncertain ecological assumptions (for example, spatially variable larval production and habitat suitability) into the model, enabling identification of potential sources, sinks and corridors across both protected and unprotected areas.

Here, we assess whether APEIs are linked through simulated larval dispersal, evaluate modelled APEI contributions to AMI replenishment under baseline and mining disturbance scenarios and identify unprotected regions capable of improving modelled network connectivity through corridor addition. Together, these analyses provide a scalable, evidence-based framework for connectivity-informed conservation planning in data-limited frontier regions.

## Results

Simulations incorporated multiple seasons, years and reasonable planktonic larval durations (PLDs) ranging from 19 to 69 days (Methods*)* to capture environmental and biological variability relevant to management-scale planning. Connectivity patterns were formally compared across scenarios (Extended Data Table 1). Results summarize connectivity integrated across all simulated conditions; values reflect relative modelled exchange within the simulation framework and should not be interpreted against absolute demographic or genetic connectivity thresholds.

### Simulated exchange among APEIs is rare and spatially restricted

**Fig. 1:**
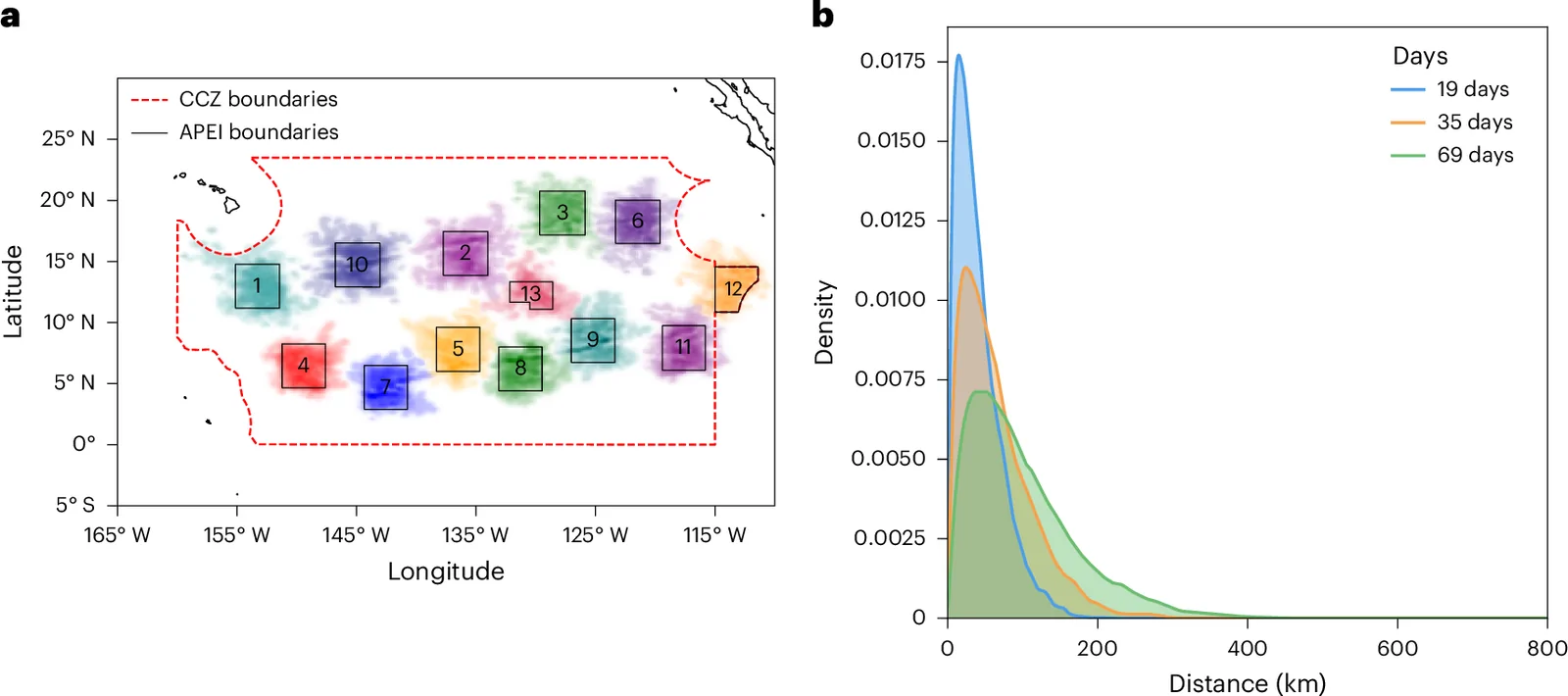
Larval dispersal footprints and dispersal-distance distributions across the CCZ. Larval dispersal characteristics across the CCZ. **a**, Cumulative near-bed larval dispersal footprints originating from each of the 13 APEIs (black outlines, numbered by official designation) across all simulated PLDs and time periods, overlaid on the CCZ management region (dashed red boundary). Footprint extent predominantly reflects the 69-day PLD. **b**, Kernel density distributions of near-bed constrained particle dispersal distances for PLDs of 19, 35 and 69 days, aggregated across all four simulation windows.

Simulated exchange among APEIs was extremely limited for PLDs up to 69 days (Fig. 1a). Across all simulations, just 0.018% of particles dispersed between APEIs under the modelled framework, while 79% remained within their origin and 21% settled outside the network entirely. Of 156 possible direct APEI-to-APEI connections, only 5 showed any exchange, dominated by three routes: APEI 5 to 8, APEI 8 to 9 and APEI 13 to 9 (cumulatively accounting for over 99% of all inter-APEI transfers). The only other direct links, APEI 2 to 13 and APEI 6 to 3, involved fewer than 15 particles each: <0.00014% of particles released from those APEIs. Two additional pathways showed potential for indirect connectivity, via sequential settlement through intermediate APEIs: APEI 2 to APEI 9 via APEI 13, and APEI 5 to APEI 9 via APEI 8. This near absence of exchange is consistent with the dispersal distance distributions shown in Fig. 1a, showing median travel distances under 100 km even at 69 days.

Self-retention of particles within the APEI from which they originated was consistently high under the grid cell resolution used in this study. APEIs 3 and 6 retained 88% and 86% of their particles, respectively, while APEI 13—the least retentive—retained 60%. Even under maximum simulated dispersal (69 days^17^), median travel distances remained under 100 km (Fig. 1b)—far below the 200–800-km gaps between APEIs^10^. For particles that failed to settle in an APEI, the median distance to the nearest non-origin APEI was still large at 389 km (interquartile range (IQR) 298–542 km), indicating that also multistep functional connectivity may remain limited under the dispersal assumptions considered.

### APEI-derived particles reach the majority of AMIs but are not the dominant modelled source

While simulated direct connectivity between APEIs is limited, modelled dispersal indicates potential exchange between APEIs and other parts of the CCZ, including designated Areas of Mining Interest (AMIs). To distinguish AMI sites receiving meaningful APEI-derived larval input from those reached by only negligibly few particles that may reflect stochastic dispersal events rather than sustained supply, we applied a minimum of 1,000 simulated particles aggregated across all simulations, an order-of-magnitude filter scaled to the ensemble size of the simulations. This threshold is not intended to represent a biological recruitment threshold but to identify AMI sites receiving substantial aggregated particle flow (Methods). Applying this threshold, 71% of AMI sites received APEI-derived particle input at 69 days PLD, reducing to 55% and 41% at 35 and 19 days, respectively (Table 1). In source-normalized terms, 4% of particles released from APEIs reached AMIs aggregated across all PLDs, indicating that the majority of APEI-released particles settle outside AMIs regardless of PLD (Table 1). Per-APEI contributions varied considerably, with APEI-13 and APEI-2 the strongest contributors aggregated across PLDs; full per-APEI breakdowns are provided in Supplementary Table 1.

**Table 1:** Modelled larval connectivity between APEIs and AMIs across simulated PLDs, reported as presence-based spatial reach (applying a minimum 1,000-particle threshold consistent with the reciprocal particle-flow threshold used in the network connectivity analysis) and proportional larval flux under baseline and mining disturbance scenarios

PLD (days)		Presence ≥1,000 particles (% AMIs)		Source-normalized (percentage APEI releases reaching AMIs)	
19		41.2		2.5	
35		55.0		4.3	
69		71.2		6.6	
Aggregated		–		4.4	
PLD (days)	Baseline: APEI origin (%)	Baseline: AMI origin (%)	Baseline: unprotected (%)	Mining-disturbance scenario: APEI origin (%)	Mining-disturbance scenario: unprotected (%)
19	2.5	81.9	15.6	14.1	85.9
35	4.5	76.5	19.1	19.0	81.0
69	6.7	70.5	22.8	22.9	77.1
Aggregated	4.6	76.3	19.1	19.3	80.7

Sink-normalized metrics partition total AMI larval settlers by origin: APEI origin, AMI origin (encompassing within-AMI retention and inter-AMI exchange) and unprotected non-mining areas, under both undisturbed baseline conditions and a mining disturbance scenario in which AMI self-supply is removed. Aggregated presence values reflect the maximum across PLDs rather than a pooled estimate, as the 69-day result is a superset of shorter PLD results. Aggregated source- and sink-normalized values are pooled across all PLDs, seasons and years.

**Fig. 2:**
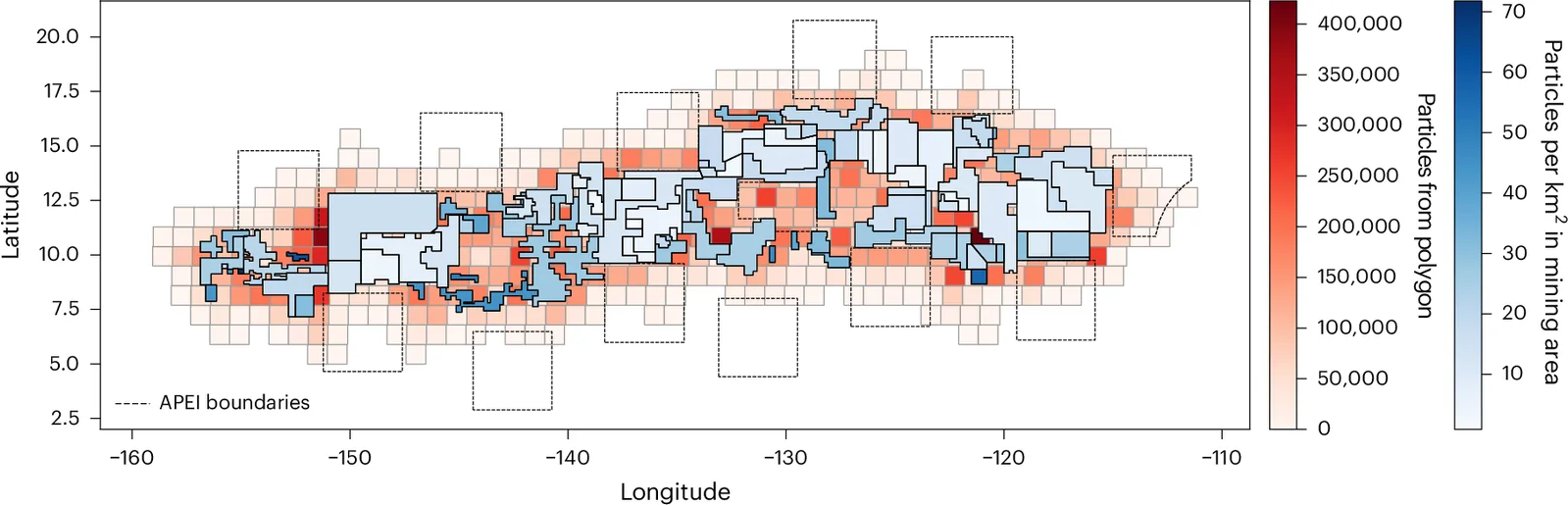
Origin and settlement densities of larvae reaching AMIs. Origin density (red) and settlement density (blue) of larval particles that settle in AMIs, released from all cells outside the AMIs (including both APEIs and unprotected, non-mining areas). Mining areas (exploration and reserved zones combined) are shown as blue polygons. Darker-blue shades indicate higher settlement densities, representing greater larval influx into mining areas. Red shading shows the density of particles originating from source cells that contributed larvae to mining areas (cumulative results for 19-, 35- and 69 days of simulated dispersal for four time periods: January 2019, July 2019, January 2023 and July 2023).

**Extended Data Fig. 1:**
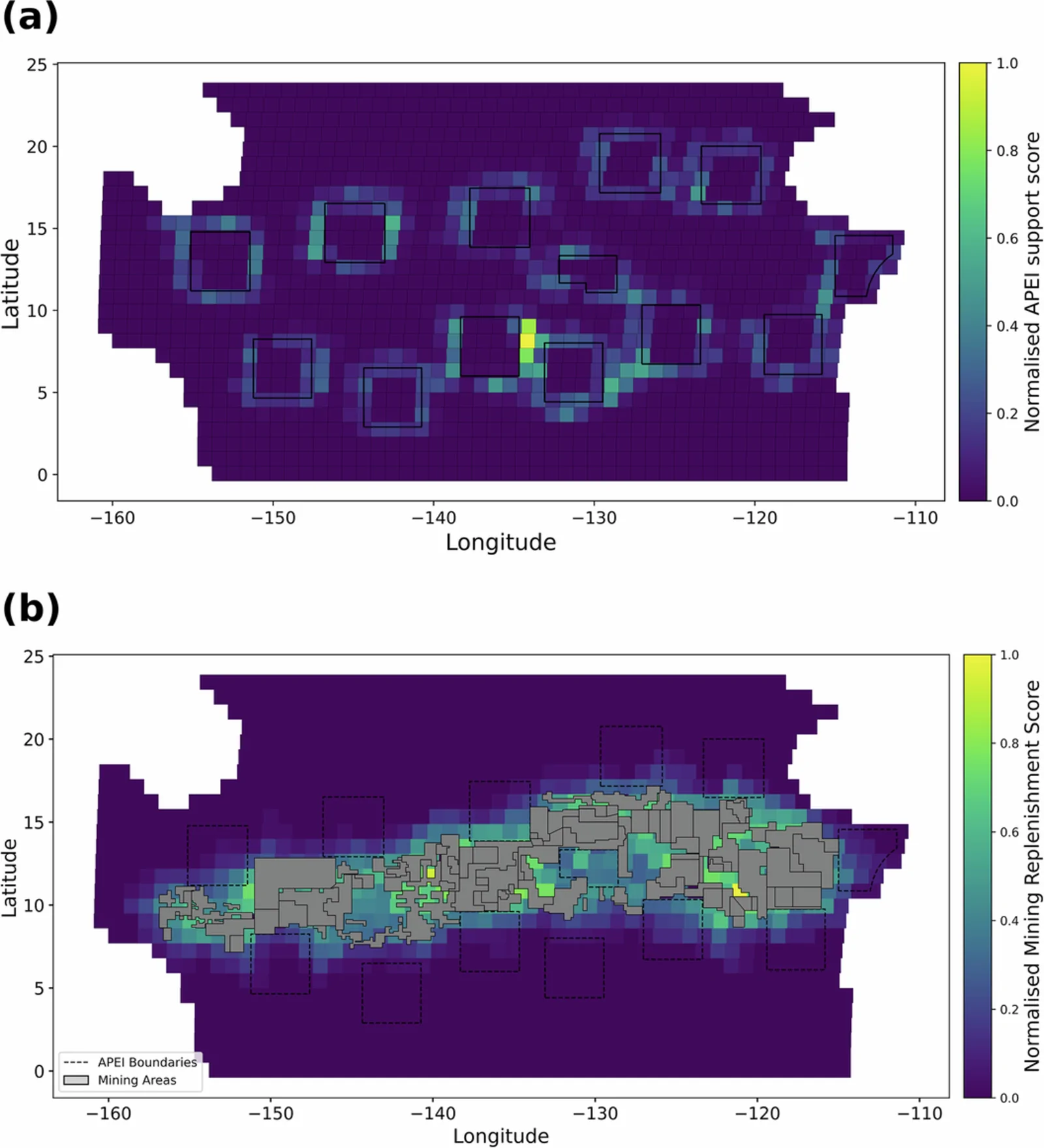
APEI support and mining replenishment scores across the CCZ. Component scores underlying the Integrated Connectivity Value (ICV) across the CCZ. Grid cells (100 × 100 km) are coloured by (top) normalised APEI support score (A), reflecting each cell’s contribution to larval connectivity across the existing APEI network, and (bottom) normalised mining replenishment score (M), reflecting each cell’s composite contribution to larval supply into mining areas based on total flux, spatial reach, and delivery efficiency. Higher scores (yellow) indicate stronger contribution in each component. Grey polygons show mining areas; dashed black outlines show APEI boundaries.

Under baseline conditions, AMIs are predominantly self-supplying: AMI-origin particles (encompassing both with AMI-retention and inter-AMI exchange) account for 76% of AMI settlers aggregated across PLDs, with unprotected non-mining areas contribution 19% and APEIs 5% (Table 1). This indicates that under undisturbed conditions, external replenishment from APEIs represents a minor component of AMI larval supply relative to internal AMI sources. Under a mining disturbance scenario in which AMI self-supply is no longer available, APEIs account for 19% of remaining AMI particle settlers aggregated across PLDs; a substantially larger relative contribution than under baseline conditions, with unprotected non-mining areas contributing the remaining 81% (Table 1 and Supplementary Table 1). Several unprotected areas, often adjacent to APEIs, emerged as dominant sources of external particle supply across both scenarios (Fig. 2 and Extended Data Fig. 1), delivering particles to multiple AMIs across the CCZ.

### CCZ connectivity is modular and locally constrained, with community structure sensitive to AMI source removal

**Fig. 3:**
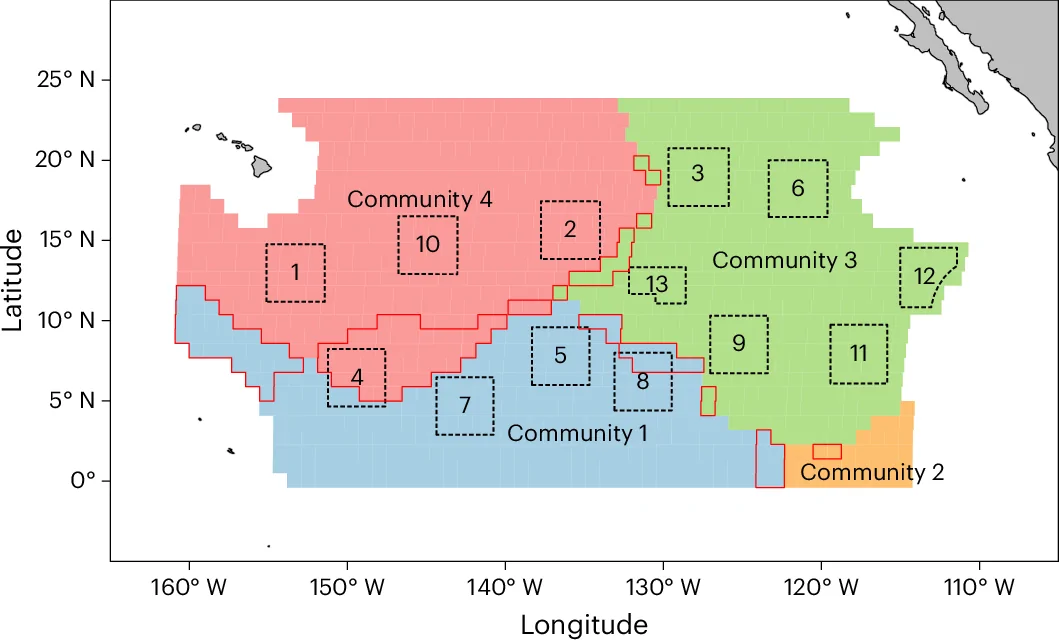
Larval connectivity communities across the CCZ and their sensitivity to mining-origin larvae. Connectivity-based network communities (calculated through graph network analysis; Methods) in the CCZ showing four distinct larval connectivity communities across the CCZ. Derived from simulated dispersal trajectories (cumulative results for 19, 35 and 69 days of simulated dispersal for four time periods: January 2019, July 2019, January 2023 and July 2023). Coloured areas represent community membership, while dashed black squares indicate the locations of APEIs. Interface zones outlined in red shift community affiliation when mining-origin larvae are removed.

**Extended Data Fig. 2:**
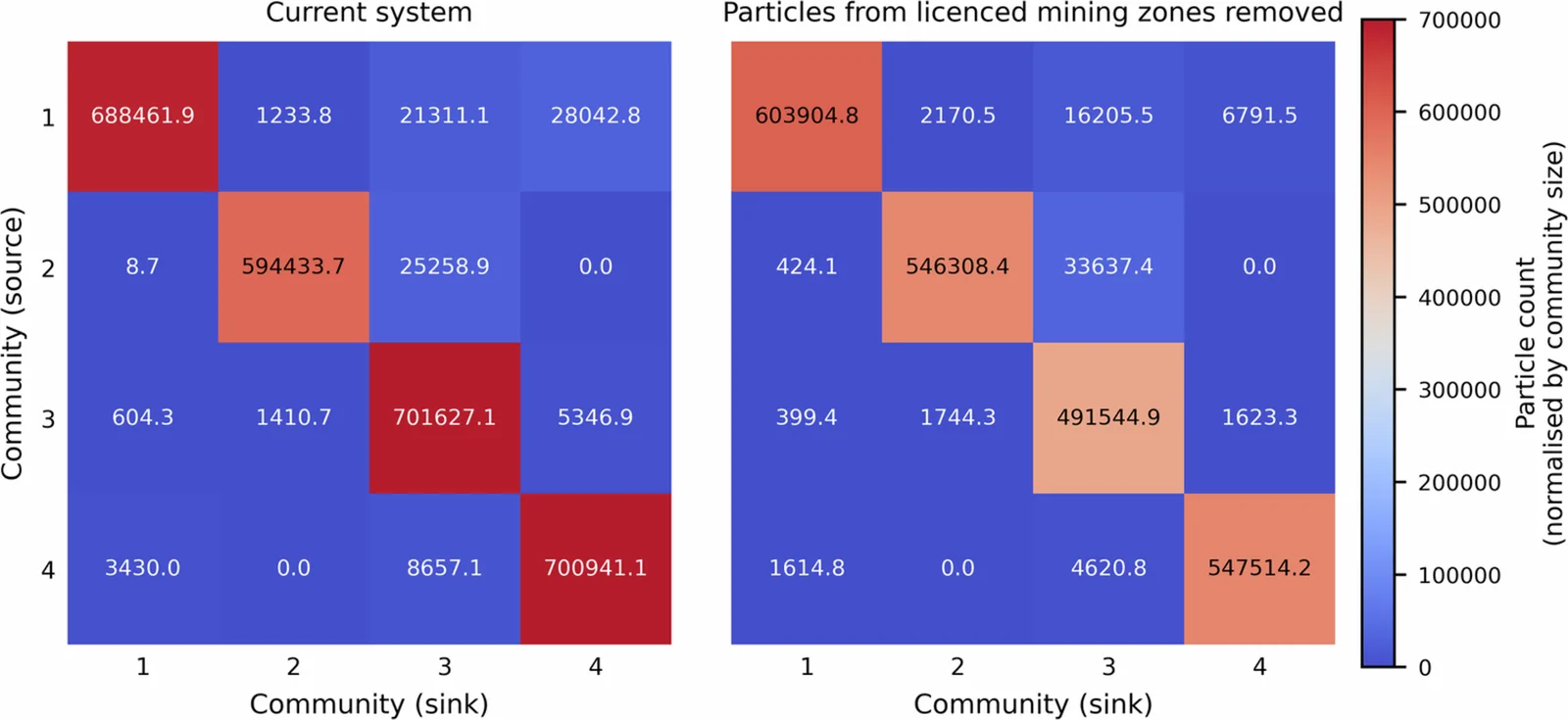
Community-level connectivity matrices showing larval exchange between connectivity communities. Community-level connectivity matrices showing the volume of larval exchange between communities, normalised by the sizes of source and sink communities. Left: current system Right: particles of mining area origin removed. Diagonal values indicate self-retention within each community, while off-diagonal elements represent cross-community exchange. Higher values (red) indicate stronger connectivity.

Simulated particle dispersal in the CCZ is strongly modular, with movement largely confined within four major connectivity communities (Fig. 3). Community 4, in the northwest, is the largest and best connected, spanning 4.41 million km (36% of the CCZ), while communities 1 and 3 together cover over 60% of the region. Despite being the smallest and containing no APEIs, community 2 displayed the highest internal connectivity. Under current conditions, modelled connectivity between communities is limited, with the majority of exchange occurring within rather than across community boundaries (Extended Data Fig. 2, left). When particles originating from mining areas are excluded in the models, representing a mining-disturbance scenario in which AMIs no longer function as larval sources, connectivity declines across the network (right panel), with both intra- and intercommunity flows reduced. Community 4 experiences particularly large losses, with a 23% reduction in outwards dispersal and 24% decline in particle input. Several regions (Fig. 3, red outline) shift their community assignment under this scenario. These areas sit at the boundaries of community partitions and appear to be structurally flexible, changing affiliation depending on modelled larval sources.

### High-connectivity cells are predominantly located outside existing APEIs

**Fig. 4:**
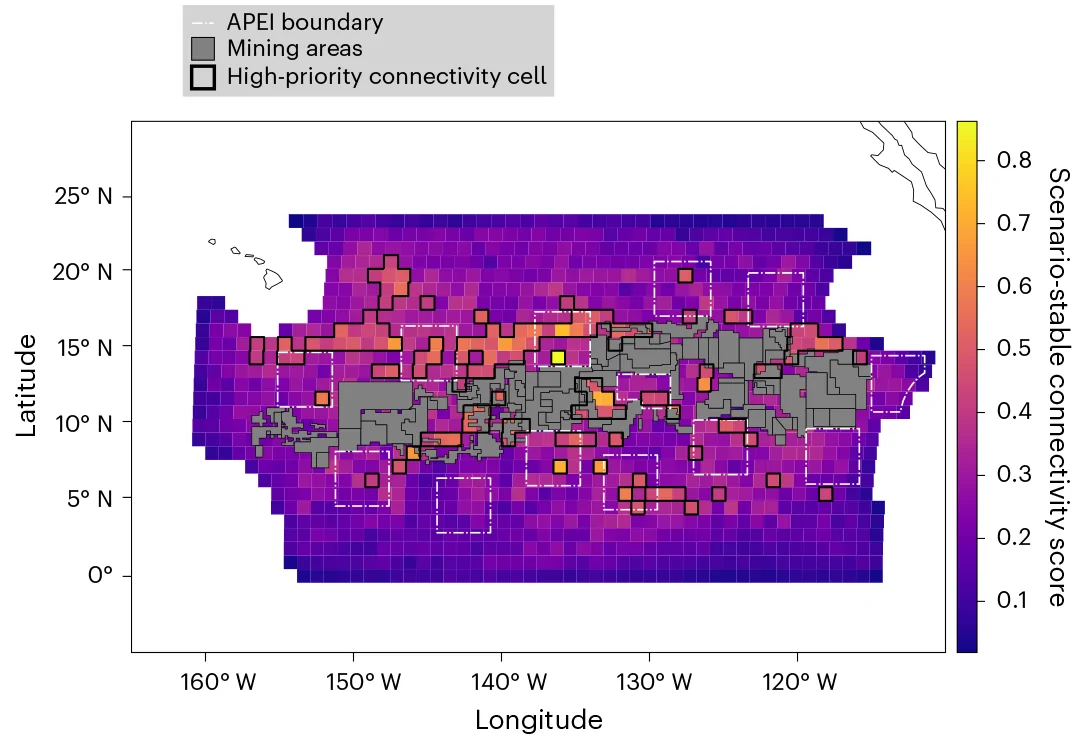
Scenario-stable composite connectivity scores across the CCZ. Scenario-stable composite connectivity score for 100 × 100-km grid cells across the CCZ, integrating source strength (out-degree) and stepping-stone potential (node betweenness) under both non-mining and post-mining scenarios (Methods). Black-outlined cells represent the top 10% of scenario-stable scores, shown for visualization purposes only; this threshold does not enter the analysis, optimization or recommendations. AMIs are shown in grey and APEI boundaries in dashed white boxes. Scores reflect each cell’s ability to support connectivity across conditions; warmer colours indicate higher, more persistent connectivity (cumulative results for 19-, 35- and 69 days of simulated dispersal for four time periods: January 2019, July 2019, January 2023 and July 2023).

Many grid cells that retain high connectivity value across both baseline and AMI source-removal scenarios occur around the AMIs (Fig. 4). The cells outlined in black represent the top 10% of locations that consistently function as larval sources and stepping stones for connectivity throughout the CCZ (not just to AMIs) in both mining disturbance and baseline scenarios, where in the ‘mining-disturbance’ scenario, the AMIs no longer function as larval sources owing to mining activities. Spatially, these high-priority cells are widely distributed, with 77% of their total area falling outside existing APEIs, and many occur along margins of AMIs, underscoring critical gaps in current spatial coverage. Their persistence across mining and non-mining scenarios suggests that they are robust features of the CCZ dispersal landscape.

### Optimized corridor additions increase inter-APEI dispersal and AMI replenishment

**Fig. 5:**
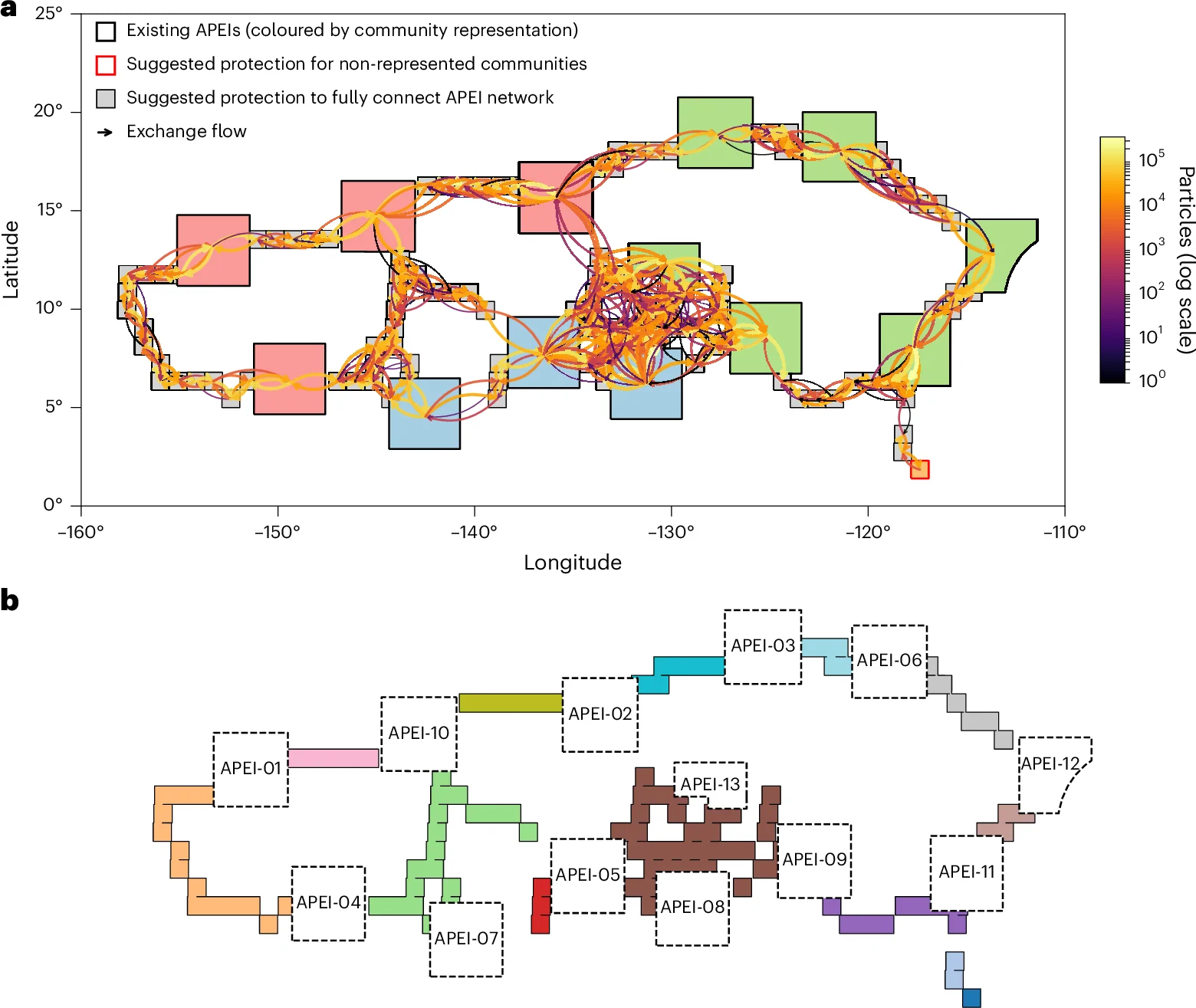
Optimized APEI network configuration for enhanced larval connectivity. **a**, Enhanced APEI network with particle exchange flows showing the optimized protected area configuration, including existing APEIs (coloured by community) and suggested additions to connect the network (black outline), and represents previously unprotected communities (red outline: one box). Arrows indicate larval exchange flows between sites, with colour intensity scaled by particle count (log_10_). **b**, The grouped configuration of the 13 sites proposed to enhance APEI connectivity (cumulative results for 19-, 35- and 69 days of simulated dispersal for four time periods: January 2019, July 2019, January 2023 and July 2023).

**Extended Data Fig. 3:**
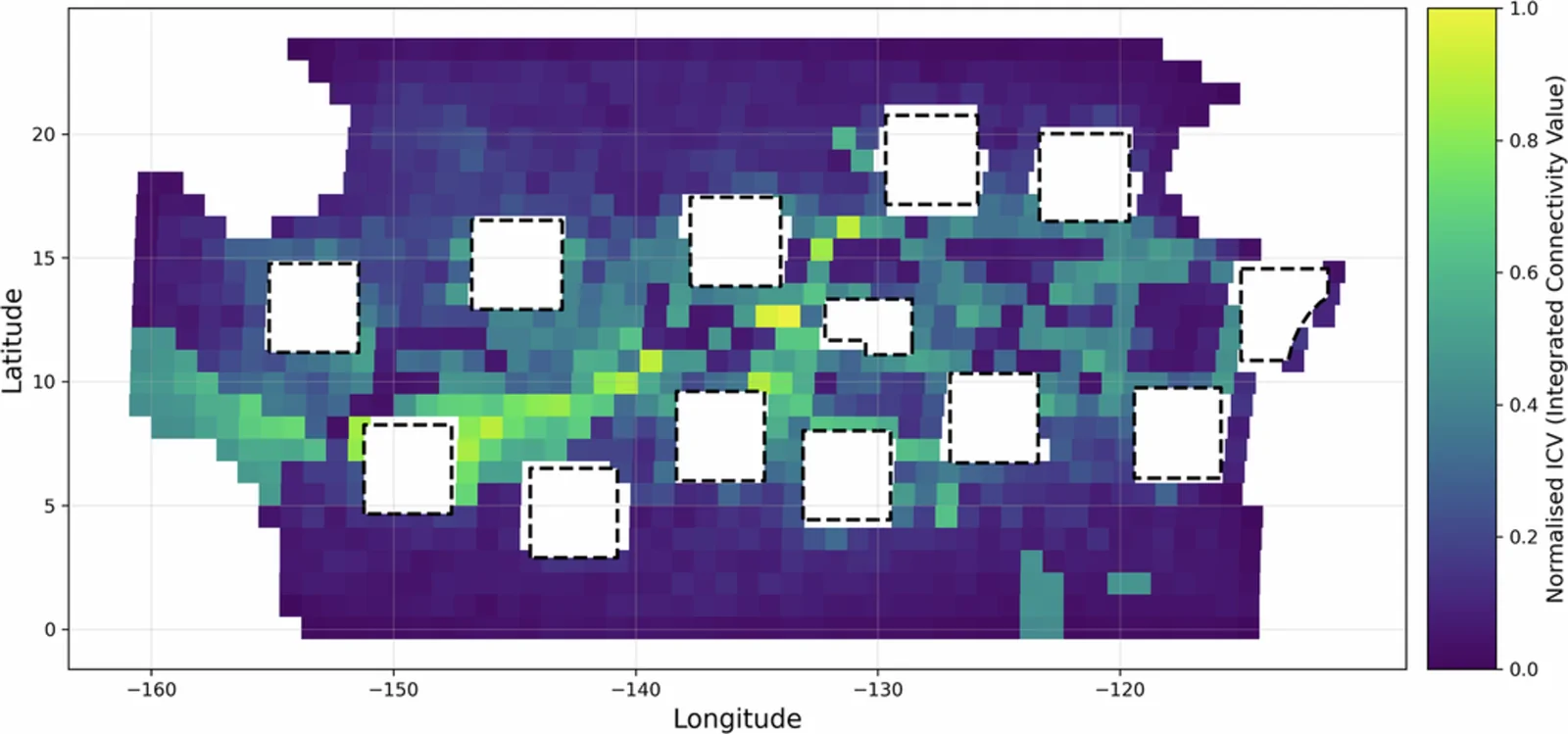
Integrated Connectivity Values (ICV) across the CCZ. Grid cells (100 × 100 km) are coloured by their normalised ICV, combining APEI support, mining replenishment potential, scenario stability, and community transition status. Dashed black outlines indicate existing APEI boundaries.

To explore how to improve the APEI system connectivity, we developed a spatial prioritization framework to identify the minimum set of new sites needed to restore connectivity between APEIs. This was achieved by establishing stepping-stone pathways between APEIs on the basis of the reciprocal dispersal link exceeding the particle-flow threshold and prioritizing cells with high integrated connectivity values (ICVs; Extended Data Fig. 3; Methods). APEIs are considered ‘connected’ when a path exists between them in this thresholded dispersal network, such that single or rare dispersal events are not sufficient to define connectivity. To connect all APEIs via the minimum number of additional sites, subject to the reciprocal particle-flow threshold, the optimization identified 53 grid cells. The resulting configuration transformed network performance (Fig. 5). While direct APEI-to-APEI links remained unchanged, these additions enabled 151 indirect pathways, increasing inter-APEI dispersal 406-fold (40,500%)—from just 1 in 5,556 particles to 1 in 14 released from protected areas reaching another protected area within the modelled network. The new sites act as corridors between current APEIs and become key stepping stones that restore connectivity between protected areas where simulated direct exchange among APEIs is limited, capturing 21% of particles from current APEIs and returning 8% back into the network, while halving the median near-miss distance of unsuccessful dispersers from 389 km to 154 km. Of the 53 corridor grid cells identified by the optimization procedure, 29 did not overlap AMIs, while 24 partially intersect the existing contract areas. In aggregate, 11% of the total proposed corridor area overlaps AMIs, with a median overlap fraction of 26% among overlapping cells, indicating retention of substantial non-mined area capable of supporting dispersal. Addition of the corridors also improves modelled connectivity to AMIs. The source-normalized contribution of the protected network to AMI replenishment increased from 4% to 8% (a doubling of supply) driven predominantly by three new sites (Fig. 5, inset: dark brown, light brown and green), which individually supply 45%, 32% and 21% of their particles to AMI cells, respectively. The proportion of AMI cells receiving sustained particle input (≥1,000 particles) from the expanded network rose from 71% to 85% aggregated across all PLDs, with gains apparent across all simulated dispersal durations and proportionally the largest at the shortest PLD (19 days: 3–6%). The value of the expanded network is the most apparent under the mining disturbance scenario: when AMI self-supply is removed, the APEI contribution to remaining AMI settlement rises from 19% to 36%, nearly doubling the backstop replenishment capacity of the protected network under disturbance and reducing dependence on unprotected non-mining sources from 81% to 64%.

## Discussion

The APEI network was designed around habitat representativity, with inter-APEI connectivity judged spatially infeasible given mining claim distributions^7,10^. Connectivity has since been recognized as an aspiration, with the 2019 Friday Harbor workshop and the 2021 expansion explicitly acknowledging the need to improve connectivity alongside representativity^11,24,25^, yet whether the network functions as a coherent connected system has never been formally assessed. Our analysis provides the first region-wide predictive assessment of potential ecological connectivity in the CCZ, on the basis of physically driven dispersal modelling. Under modelled larval dispersal conditions, the network has limited capacity to support exchange between protected areas, suggesting it may not function as an ecologically coherent unit and consequently may fail to establish functional connectivity standards essential for ecological resilience^20,22,26^. Although APEIs contribute to particle supply across the majority of mining areas, AMIs are predominantly self-supplying under undisturbed conditions, and it is unprotected non-mining areas that emerge as the dominant modelled source of larval supply, with implications for how conservation value may be distributed across the wider CCZ.

Modelled dispersal is further structured into four semi-isolated connectivity communities, within which a small number of unprotected regions contribute disproportionately to maintaining exchange—areas whose conservation status may have implications for both AMI replenishment and the broader structural integrity of the region. Strategic corridor addition resolves much of this structural deficit, transforming a largely disconnected protected area system into one capable of sustaining indirect exchange across the full CCZ, with benefits most pronounced under mining disturbance. Under modelled conditions, the identified additions would, if protected, provide the stepping stones needed to functionally connect the existing APEI system and substantially improve AMI replenishment from protected areas. The corridors identified here are not intended as substitutes for these core protected areas—large APEIs remain important for protecting representative habitats and supporting viable, self-sustaining populations across the CCZ^4^—but as complementary additions with the potential to enhance exchange among APEIs and between protected and impacted regions. Effective regional conservation will therefore depend on maintaining both well-connected corridors and sufficiently large, protected areas capable of sustaining populations over long timescales.

The near-absence of modelled inter-APEI exchange in the CCZ is consistent with the spacing constraints identified at the time of design^10^: modelled dispersal distances confirm that the 400–800-km inter-APEI gaps exceed what passive near-bed transport can bridge within biologically plausible timeframes^17^, leaving individual protected areas dependent on self-recruitment rather than exchange with neighbouring sites^27^. Modelled dispersal distances under near-bed transport are broadly consistent with empirical evidence that realized dispersal in marine invertebrates is frequently shorter than theoretical expectations^28^, suggesting that limited inter-APEI exchange may reflect genuine dispersal constraints rather than an artefact of the modelling framework. The dominance of unprotected areas as modelled particle sources under disturbance conditions is in part a geometric consequence of how the APEIs were originally placed: designed for habitat representativity and broad ecological resilience rather than larval replenishment of mining areas. Their positions were constrained by pre-existing exploration contracts^7,9,10^, leaving central unprotected areas better positioned hydrodynamically to supply AMIs under near-bed circulation. APEIs contribute a small share of modelled AMI larval supply under undisturbed conditions, with their relative importance increasing substantially when AMI self-supply is removed by disturbance, though unprotected non-mining areas remain the dominant external source throughout. That unprotected areas dominate modelled AMI supply under disturbance is therefore unsurprising, however many of the same central areas also disproportionately exhibit high scenario-stable connectivity scores, suggesting they play an outsized role in the modelled regional exchange that ecological resilience across the CCZ may depend on. The lack of formal protection for these areas therefore warrants consideration within the resilience framework the APEIs were explicitly designed to deliver.

The modelled subdivision of the CCZ into four semi-isolated connectivity communities is consistent with emerging evidence that the region comprises distinct biogeographic regions rather than being a single homogeneous system^5,29^. Population genetic structure in CCZ fauna further supports regional-scale subdivision^30^, although genetic clusters do not map directly onto the four modelled communities, as expected given that genetic structure integrates species-specific biology and historical processes over longer timescales than circulation-driven dispersal potential. One modelled community falls entirely outside the current protected network, illustrating how connectivity-defined regions can fall outside representativity-based frameworks, although its management relevance depends on current and future pressures. Notably, some areas shift modelled community affiliation under mining disturbance, suggesting that regional connectivity structure may be reorganized when AMI larval sources are removed. These transitional zones sit at community boundaries and their conservation status may therefore have implications not just for AMI replenishment but for maintaining the broader structural integrity of the modelled regional network.

Interpreting these results requires awareness of several important constraints. As with all biophysical dispersal models, the outputs presented here represent a simplification of a complex system, capturing dominant physical drivers of larval transport under stated assumptions rather than fully replicating the biological and oceanographic complexity of the real environment. The analysis therefore evaluates structural dispersal pathways rather than demographic sufficiency: addressing whether the APEI system forms a structurally coherent dispersal network under plausible assumptions (a question that can be answered with available data), rather than whether connectivity is sufficient to ensure population persistence (which cannot). These constraints are by design rather than oversight: in a system characterized by extreme data scarcity, a transparent, physically grounded baseline is more informative for planning purposes than a complex model embedding poorly constrained biological assumptions. Connectivity patterns are aggregated across four simulated time periods spanning seasonal and interannual variability and across three PLDs representing a plausible range for deep-sea taxa (Extended Data Table 2); this provides an integrated representation across simulated conditions rather than a formal uncertainty analysis. It should be noted, however, that these time periods do not encompass the full range of El Niño–Southern Oscillation (ENSO) variability (Extended Data Table 3) nor longer-term decadal circulation patterns; results should be interpreted as integrating the circulation states of the four time intervals modelled rather than as long-term climatological averages.

**Extended Data Fig. 4:**
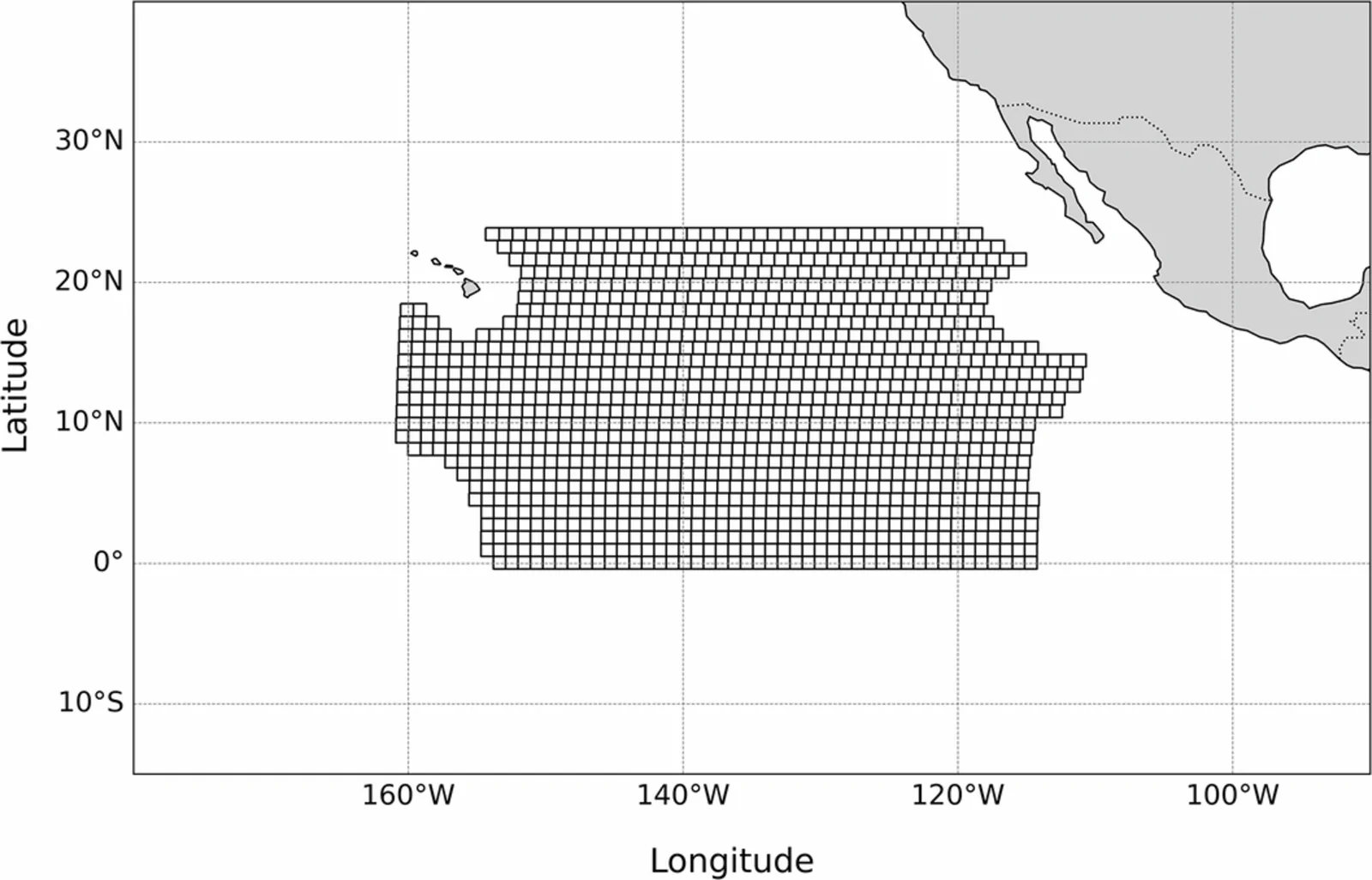
100 × 100 km grid cells (N = 1,225) covering the area of the Clarion-Clipperton Zone (CCZ). Grid map showing the spatial framework used for particle release and connectivity analysis.

All estimates should accordingly be understood as conditional outputs under a consistent physical framework: HYCOM provides the best-available basis for regional-scale abyssal inference, but direct validation at these depths remains sparse, and unresolved tidal processes and fine-scale bathymetry may influence near-bed transport and within-cell diffusivity locally^31^, meaning inferences are most robust at the 100 km × 100 km planning scale (Extended Data Fig. 4). PLD data for CCZ taxa are extremely sparse, and the three durations modelled represent a pragmatic, taxa-agnostic range; the finding of limited inter-APEI exchange is robust across all three within the near-bed transport framework, lending confidence to the core conclusions despite this uncertainty. In reality, some taxa may disperse for longer than the modelled range; however, extending beyond this range within the model would compound existing uncertainties in larval survival, behaviour and competency, none of which are empirically constrained for abyssal CCZ taxa at any PLD, risking a progressive amplification of error that the modelling framework cannot currently account for.

**Extended Data Fig. 5:**
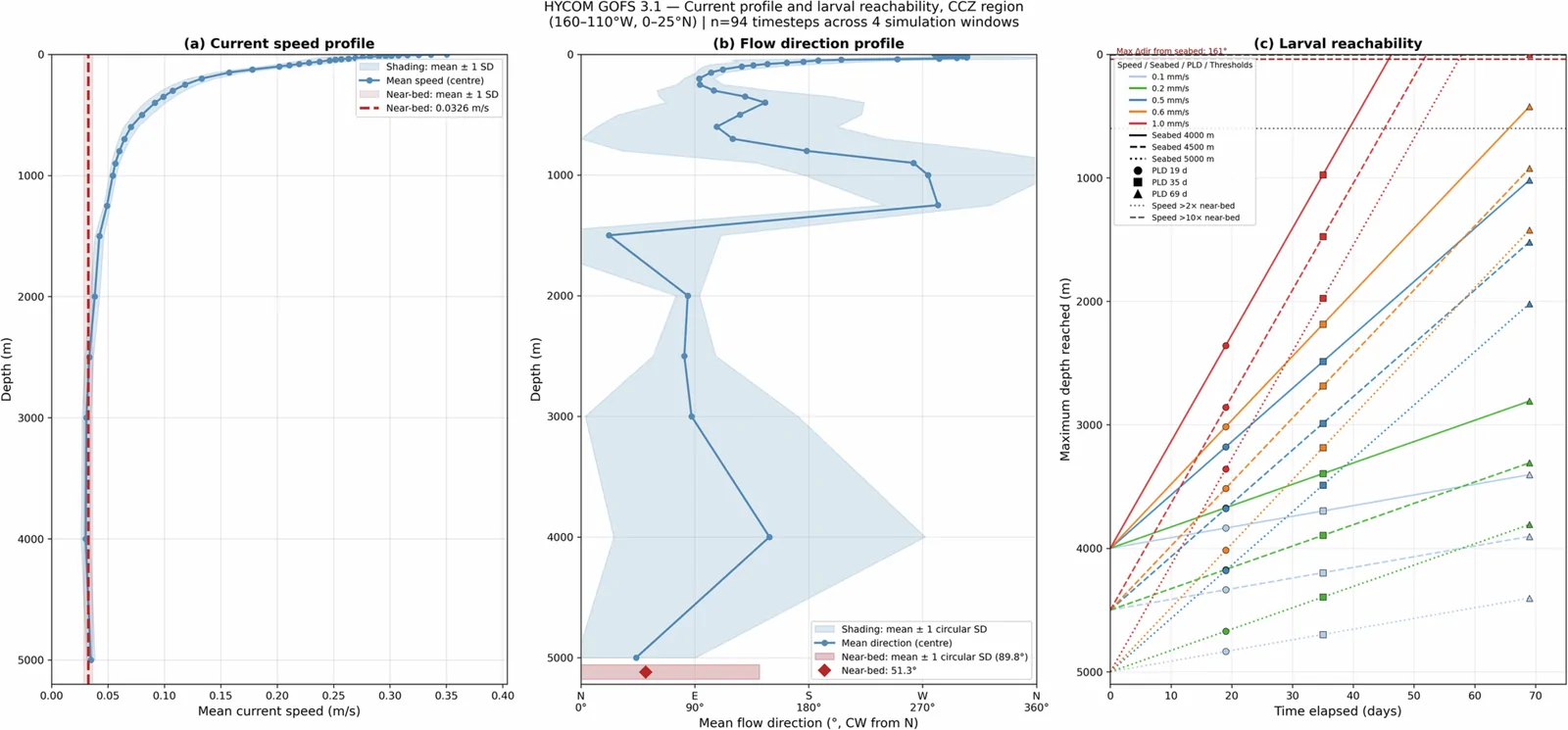
HYCOM velocity profile and larval reachability analysis for the CCZ region. HYCOM GOFS 3.1 velocity data sampled every three days across all four simulation windows (n = 94 timesteps; 160–110°W, 0–25°N). (**a**) Mean current speed with depth; data are presented as mean values and the shaded band denotes ± 1 s.d.; the red dashed line indicates the near-bed speed (mean 0.0326 m s^−1^) and the red shaded span its ± 1 s.d. (**b**) Mean flow direction with depth; data are presented as mean values and the shaded band denotes ± 1 circular s.d.; the red diamond indicates the near-bed direction (mean 51.3°) and the red shaded span its ± 1 circular s.d. (89.8°). (**c**) Maximum depth reached under continuous upward swimming as a function of time elapsed, for swimming speeds of 0.1–1 mm s^−1^ and CCZ seabed depths of 4000, 4500 and 5000 m; values follow a deterministic kinematic relationship (no error statistic applies). Horizontal reference lines indicate depths at which mean current speed exceeds 2× and 10× near-bed speed.

Vertical behaviour was not included in the model; constraining particles to near-bed flow is a specific assumption about larval vertical position rather than an absence of assumption. In the absence of empirical constraints on vertical behaviour for abyssal CCZ taxa, introducing additional behavioural parameters risks compounding rather than reducing model uncertainty^32^. Given the species-agnostic framework, species-specific behavioural parameterization would in any case be inappropriate. Empirical analysis of HYCOM velocity profiles (Extended Data Fig. 5) demonstrates that near-bed confinement is demonstrably conservative with respect to dispersal distance for taxa swimming within empirically bounded speed ranges (Methods), but flow direction varies substantially with depth, meaning taxa with sustained upward swimming capacity could access faster currents in substantially different directions—dispersal pathways not captured by this framework and requiring species-specific data and targeted modelling as empirical constraints accumulate. Reported dispersal patterns in this study should therefore be interpreted as conditional on the near-bed depth constraint rather than as upper bounds on dispersal potential for all CCZ taxa.

The analysis focuses on larval dispersal only, which is appropriate given the predominance of sessile or weakly motile CCZ taxa but means results are most directly applicable to species with pelagic larval stages; while corridors may provide indirect benefits for taxa with limited or no pelagic dispersal through habitat continuity and protection of stepping-stone environments, this cannot be evaluated within the present framework.

The uniform release assumption means habitat quality is not reflected in source weighting; particulate organic carbon-scaled or habitat-weighted larval production could shift corridor priorities for particular taxa, without altering the core finding of limited direct inter-APEI exchange, and if connectivity is limited under uniform release, biological or environmental constraints are unlikely to strengthen it. Corridor outputs should therefore be evaluated alongside habitat, nodule density and carbon flux data as complementary planning layers. The mining scenario is source-loss only and does not capture sediment plumes or acoustic disturbance beyond contract boundaries; where such impacts do extend further, wider buffers may be warranted, although empirical constraints on plume extent in the CCZ are currently insufficient to prescribe specific dimensions^33^. Sediment plumes in particular could increase near-bed larval mortality beyond contract boundaries^34^, representing an additional mechanism by which the disturbance scenario may understate connectivity impacts; the source-removal scenario should therefore be interpreted as a conservative lower bound on mining-related connectivity disruption rather than a complete representation of disturbance effects. Taken together, these constraints do not undermine the study’s core contributions—the outputs provide a reproducible, physically grounded basis for connectivity-informed spatial planning that can be refined as empirical data become available.

The CCZ APEI network, as currently configured, does not function as a coherently connected system under modelled larval dispersal conditions, but this is a structural limitation that targeted corridor addition could meaningfully address. The extent to which network performance improves when intermediate sites are added suggests both the potential vulnerability of the current configuration and that meaningful structural improvement may be practically achievable. Refining these projections will require the empirical research that connectivity models of this kind depend on: near-bed circulation observations in predicted linking zones, population genomic sampling across modelled community boundaries, improved constraints on larval behaviour and PLD for representative CCZ taxa and habitat-weighted scenarios incorporating nodule abundance and particulate organic carbon flux would collectively enable iterative refinement of connectivity estimates and corridor priorities while providing the field and biological validation currently absent for abyssal dispersal modelling. We advocate embedding connectivity into conservation design, refining projections as this evidence accumulates, and acting to reduce the risk of long-lasting damage to the abyssal ecosystem before commercial mining at scale proceeds (for policy recommendations and a relevance summary, see https://doi.org/10.5281/zenodo.21413810 (ref. ^35^) and the Supplementary Information).

## Methods

### The study area

A uniform 100 km × 100 km (10,000 km^2^) grid was generated across the CCZ region (160°–110° W, 0°–25° N), balancing spatial resolution with computational feasibility and alignment with the scale at which APEIs and AMIs are defined and managed, and is treated as an explicit design choice motivated by management relevance rather than ecological optimality. Each 200 km × 200 km APEI with 100-km buffers spans approximately 16 grid cells at this resolution, providing adequate coverage for network-level connectivity assessment. The analysis is not designed to resolve fine-scale spatial structure within individual APEIs; doing so would require higher-resolution circulation data and substantially greater computational effort and is beyond the scope of this study. Metrics derived from this grid are therefore inherently scale dependent and should be interpreted comparatively across planning units, rather than as absolute estimates of biological retention. Connectivity patterns should therefore be interpreted at the regional planning scale; processes operating at finer spatial scales, such as localized bathymetric steering or individual seamount-scale retention, are not resolved by this framework. To account for Earth’s curvature, latitude and longitude offsets were adjusted per cell size. Land polygons from Natural Earth^36^ were used via the ‘cartopy’ package^37^ to exclude terrestrial cells. The grid was clipped to the CCZ management area^38^, resulting in 1,225 ocean-only cells (Extended Data Fig. 4).

### The biophysical model

#### The hydrodynamic model

The GOFS 3.1 HYCOM model^39^ provides 41-layer global ocean circulation data at 0.08 × 0.04° horizontal resolution (~9 km × 4.5 km). Zonal (‘water_u_bottom’) and meridional (‘water_v_bottom’) bottom-layer velocities were retrieved at 3-h intervals for 170–110° W, 5° S to 25° N, spanning January 2019 to September 2023. Spatial bounds extended 10° latitude and 5° longitude beyond the CCZ to capture full particle trajectories within their PLDs. Bottom velocities represent flow 10 m above the sea floor and follow GEBCO-derived bathymetry^40^.

HYCOM GOFS 3.1 is a formally validated, operationally deployed modelling framework^41^, routinely assessed against satellite altimetry, sea surface temperature and subsurface profiles from Argo floats and moored buoys. However, this validation does not extend to bottom velocities at abyssal depths, where direct observational data for independent assessment are absent across the CCZ—a constraint common to all current global ocean models rather than a limitation specific to HYCOM. Several additional limitations apply to its use in deep-sea settings. Bottom boundary layer processes are parameterized^42^ but may miss near-bed turbulence and fine-scale dynamics. Resolution limits topographic steering and fails to resolve small bathymetric features. Together, these factors smooth the simulated circulation field, weakening retention features such as topographically steered gyres and boundary layer trapping. As a result, particles may appear to disperse more widely than they would under finer-scale flow, leading to potential overestimation of connectivity between communities and underestimation of local retention. Episodic events such as eddy-driven current reversals^43^ are also not captured, meaning rare but ecologically important long-distance dispersal may be under-represented. HYCOM does not explicitly resolve barotropic tidal processes or internal tides at abyssal depths; while tidal currents may influence near-bed transport and within-cell diffusivity locally^31^, the analysis targets broad-scale connectivity at ≥100 km resolution, at which scale these unresolved processes are unlikely to reverse the qualitative patterns identified. Taken together, these limitations mean that the connectivity estimates produced by this study should be interpreted as conditional outputs under a consistent physical framework rather than empirically validated rates of exchange; their primary utility is comparative, evaluating relative connectivity and network structure across planning units rather than providing absolute predictions of dispersal. The results are the most reliable at spatial scales comparable to the 100 km × 100 km connectivity grid used in this study, and circulation patterns at smaller spatial scales, particularly below several tens of kilometres where bathymetric features such as seamounts and ridges influence near-bed flow, may not be fully resolved.

#### Lagrangian particle tracker

Throughout this study, ‘particles’ refers to modelled Lagrangian tracers used to represent the dispersal of larval propagules; ‘larvae’ is used when referring to the biological entities these tracers represent. Particle tracking was performed using Ocean Parcels v2.1^44^, forced by the hydrodynamic model. Simulations were run for January and July in both 2019 and 2023 to capture inter- and intra-annual variability, including El Niño/La Niña phases^45–48^ (Extended Data Table 3). Particles were released every 25 km across the CCZ^23^, yielding 18,679 unique release sites (∼16 per 100 km × 100 km cell). Particle positions were recorded at 19, 35 and 69 days, representing the lower interquartile boundary, median and upper interquartile boundary of pooled eurybathic and deep-sea PLDs, respectively^17^, using a 2D fourth-order Runge–Kutta advection scheme with stochastic diffusion (see the ‘Diffusivity’ section). Dispersal distances were estimated as great-circle distances between release and settlement points using the haversine formula, accounting for Earth’s curvature^49^. While not reflecting exact trajectories, this provides a reliable measure of dispersal extent for assessing deep-sea connectivity patterns.

Trajectories were simulated for up to 69 days, following the recommendation of Hilario et al.^17^, consistent with the recommendation that MPA planning in the deep sea should use a PLD that guarantees a minimum dispersal distance for a wide range of species^17^, and their finding that 69 days satisfies the criterion for 75% of eurybathic and deep-sea species in their cross-taxon synthesis. Hilario et al. note that high variability between and within taxonomic groups and the sparsity of deep-sea PLD data limit broader generalizations. We acknowledge that longer PLDs have been recorded for some deep-sea taxa, whose dispersal potential may exceed the modelled range^17^; however, the conclusions of this study are explicitly conditional on the modelled framework and do not claim to represent the full biological diversity of dispersal strategies of organisms in the CCZ. Empirical PLD data for CCZ nodule fauna specifically are absent and this absence itself reinforces the rationale for a species-agnostic approach: in the absence of taxon-specific data, a cross-taxon synthesis provides a more defensible and representative basis for regional planning than any single-taxon parameterization would. Using PLDs that extend beyond the 75th percentile of this distribution risks skewing a species-agnostic planning framework towards the dispersal capacity of a small minority of potentially atypical long-ranging taxa, producing outputs unrepresentative of the broader community.

Particles were constrained to the bottom layer (10 m above the sea floor), reflecting empirical observations that meroplankton in the CCZ are concentrated within the benthic boundary layer, with higher larval fluxes recorded within tens of metres of the seabed than at greater heights above the bottom^50^. Constraining particles to near-bed flow is a specific assumption about larval vertical position rather than an absence of assumption. To assess the physical consequences of this choice, we examined HYCOM velocity profiles across the CCZ every 3 days across all four simulation windows (94 instantaneous snapshots), comparing terrain-following near-bed velocities (10 m above seabed) against full water column profiles at all 40 HYCOM depth levels (Extended Data Fig. 5).

##### Current speed

Near-bed speeds averaged 0.033 ± 0.004 m s^−1^—among the lowest in the water column (Extended Data Fig. 5a). Speeds throughout the 2,500–5,000-m depth range are essentially equivalent to or marginally slower than near-bed (ratio 0.93–1.03×), only beginning to increase appreciably above 2,000 m (1.18×), reaching 1.5× near bed at 1,250 m, 2× at 600 m and approximately 11× at the surface. Near-bed confinement is therefore conservative with respect to dispersal distance, although the degree of underestimation depends on vertical swimming capacity.

##### Flow direction

Flow direction varies substantially with depth across the CCZ (Extended Data Fig. 5b), and unlike current speed, near-bed confinement cannot be characterized as conservative with respect to flow direction—vertical swimming could expose larvae to fundamentally different directional regimes, potentially enabling dispersal pathways not captured by this framework. This represents a genuine limitation of the near-bed baseline and one that cannot be resolved without empirical constraints on larval vertical positioning behaviour that do not currently exist for CCZ nodule fauna.

##### Larval reachability

Larval vertical distribution in abyssal taxa remains poorly constrained^50^, representing a fundamental knowledge gap that limits parameterization of vertical behaviour in any deep-sea dispersal model^17,51^. No empirical swimming-speed measurements exist for CCZ nodule fauna specifically; among the few available for any deep-sea larvae are 0.2–0.6 mm s^−1^ for vent polychaetes^52^, and ~0.5 mm s^−1^ for cold-water coral larvae at 200–1,000 m depth^53^, neither of which are directly applicable to abyssal CCZ nodule fauna.

At the lower speeds (0.2 mm s^−1^), larvae from CCZ seabed depths (4,000–5,000 m) would reach 2,808–3,808 m within 69 days under continuous sustained swimming—depths at which current speeds are essentially equivalent to near bed (ratio 0.93–0.95×; Extended Data Fig. 5c), confirming near-bed transport as a reasonable proxy for taxa swimming at or below this speed within the modelled PLD range. At the upper bound (0.6 mm s^−1^) and with the same PLD, larvae could theoretically reach 423–1,423 m from seabed depths of 4,000–5,000 m where speeds are 1.3–2.8× faster. For taxa with both vertical swimming capability and PLDs exceeding the 75th percentile of the Hilário et al. distribution^17^—which exist, and whose prevalence among deep-sea species remains uncertain given the sparsity of available PLD data—surface currents running approximately 11× faster than near bed in an opposing direction could enable entirely different dispersal pathways from those modelled here. Critically, however, all reachability estimates assume continuous sustained upwards swimming throughout the full PLD: biologically unrealistic for any larva. In practice, larvae alternate between swimming bouts, passive sinking and feeding, meaning that within a given PLD, a larva would either reach shallower depths than the theoretical maxima or take substantially longer to reach any given depth—in either case, reducing the time spent in faster mid-water currents and limiting the dispersal advantage of any vertical migration. In addition, ascending larvae would encounter progressively warmer temperatures and lower pressures—conditions of unknown tolerance for CCZ nodule fauna and a potential physiological barrier independent of swimming capacity.

Reported dispersal distances consequently reflect bottom circulation alone and should be interpreted as conditional on the near-bed depth constraint rather than as upper bounds on dispersal potential for all CCZ taxa. Near-bed confinement using the terrain-following velocity fields at 10 m above seabed therefore represents not only the most physically precise and biologically defensible baseline currently available for a species-agnostic framework in this data-limited environment. As such, this paper addresses a specific question: whether the APEI network functions as a structurally connected system under near-bed passive transport across a biologically grounded PLD range. The framework is explicitly designed as a reproducible baseline to be refined through species-specific analyses as empirical data for CCZ taxa accumulate^54^.

#### Diffusivity

To simulate subgrid-scale horizontal movement, we implemented a stochastic diffusion scheme using the Smagorinsky method^55^ within Ocean Parcels. Horizontal eddy diffusivity was calculated from local velocity gradients using a Smagorinsky constant (*C*_s_ = 0.1) and a central difference approach with a spatial offset (d*x* = 0.01°). These values follow the standard implementation of the Smagorinsky diffusion scheme in Ocean Parcels and are consistent with established practice in offline Lagrangian connectivity studies using coarse-resolution global circulation models^44^. Velocity components (*U*, *V*) were extracted at ±d*x* in both longitude and latitude to compute gradients: d*u*d*x,* d*u*d*y,* d*v*d*x* and d*v*d*y*. These were used alongside the area of the computational cells (*A*) to calculate horizontal diffusivity (*K*_h_) as

$${K}_{{\rm{h}}}={C}_{{\rm{s}}}\times A\sqrt{{\rm{d}}u{\rm{d}}{x}^{2}+0.5\times {({\rm{d}}u{\rm{d}}y+{\rm{d}}v{\rm{d}}x)}^{2}+{\rm{d}}v{\rm{d}}{y}^{2}}.$$

Stochastic displacement was drawn from a normal distribution based on *K*_h_ and the model timestep (random walk)^56^, simulating variability from turbulent flow, and added to the particles position. A fixed random seed ensured reproducibility, isolating hydrodynamic differences between simulations from random variation.

#### Sensitivity analysis

Several tests were undertaken to identify the optimal internal model timestep and optimal number of particles released from each site for accurate representation and computational feasibility.

##### Internal timestep

To ensure numerical stability and accuracy, we applied the Courant–Friedrichs–Lewy (CFL) condition^57^, which requires that particles travel less than one grid cell per timestep. This is expressed as

$${CFL}=\frac{U\times \Delta t}{\Delta x}\le {C}_{\max }$$

where *U* is the maximum current velocity (in metres per second), Δ*t* is the hydrodynamic model timestep (in seconds), Δ*x* is the spatial resolution of the model grid (in metres) and *C*_max_ is 1 for model stability. At the hydrodynamic model’s native 3-h timestep and resolution, the CFL number was 0.509—within stable limits. However, as it is suggested to use an internal timestep that is less than the temporal resolution of the underlying model in particle tracking studies to capture the dynamics of larval interactions with hydrodynamic features^58,59^, we adopted a 1-h internal timestep, reducing the CFL to 0.2 and improving model fidelity.

##### Optimal particle number

To determine an optimal number of particles per release site (PPS), we ran simulations for 69 days with PPS values ranging from 100 to 10,000. For each run, we recorded particle start and end cell IDs and calculated the number of unique cell-to-cell connections. We then fitted a logarithmic model using SciPy’s ‘curve fit’ function^60^ to describe the relationship between the PPS and the number of unique connections, capturing the pattern of diminishing returns (Supplementary Information). To quantify this effect, marginal return was calculated as the change in unique connections per 100 additional particles per site. This approach aligns conceptually with Simons et al.^61^, who used variance-based thresholds, but here, a curve-fitting method was used to suit scalar connectivity data and improve computational efficiency. The point of diminishing returns was defined as a marginal gain of fewer than one unique connection per additional 100 PPS, reached at ~400 PPS. A ‘flatline’ point—where gains fell below 0.1 unique connections per additional 100 PPS—was identified at ~3,600 PPS. To ensure compatibility with parallel processing, this was rounded up to 3,648 PPS, yielding 68,140,992 particles per simulation across all 1,225 release sites.

#### Accounting for variation

To capture seasonal and interannual variability, we ran simulations for four timepoints (Extended Data Table 2). The four periods sampled were selected to contrast seasonal extremes and different ENSO backgrounds within the available HYCOM archive, and results were aggregated across periods to reduce sensitivity to any single circulation state. However, this sampling does not encompass the full range of ENSO variability (which operates on 2–7-year cycles) nor longer-term decadal circulation patterns. Results should therefore be interpreted as conditional on the circulation states sampled rather than as long-term climatological averages.

Particle positions were recorded at 19, 35 and 69 days as described above. We then calculated Jaccard dissimilarity^62^ for each pairwise comparison (for example, January 2019 versus July 2019) to assess differences in dispersal pathways, on the basis of binary representations of cell-to-cell connections, and used Cohen’s Kappa^63^ to quantify similarity in connectivity patterns. To test whether observed between-period Jaccard similarity was lower than expected under random reshuffling, we performed a permutation test by shuffling one array 999 times to generate a null distribution (Extended Data Table 1). As the pairwise comparisons indicated that connectivity patterns differed across seasons and years (Extended Data Table 1), all simulations were pooled before connectivity analysis to provide an integrated view that captures this seasonal and interannual variation.

Connectivity metrics are presented as aggregated values across all simulated periods and PLDs rather than as point estimates with formal error bounds. This reflects the design of the analysis as an aggregated baseline characterization of potential connectivity under defined modelling assumptions, rather than a statistical estimation exercise. Uncertainty in this framework arises principally from incomplete knowledge of near-bed circulation, larval behaviour and settlement—sources that cannot be meaningfully quantified through bootstrap or Monte Carlo approaches without introducing additional poorly constrained assumptions. Velocity-perturbation sensitivity analysis was not undertaken, as abyssal hydrodynamic uncertainty is dominated by spatial structure and unresolved mesoscale processes rather than uniform magnitude errors; implementing physically meaningful uncertainty ensembles would require alternative circulation products not currently available for the CCZ at appropriate resolution. Dispersal distance distributions across individual PLDs are shown in Fig. 1a, and pairwise statistical comparisons of connectivity patterns across simulated periods are reported in Extended Data Table 2; these are intended to illustrate the range of outcomes and assess robustness of the core findings rather than to define statistical confidence intervals.

### Connectivity assessments

To assess both the interconnectedness of the existing APEI network and the broader-scale connectivity patterns across the CCZ, the particle tracking simulation outputs were used to perform a number of analyses.

#### APEI network functionality

We first isolated particles released from within APEIs, allowing assessment of retention and exchange within the designated protected areas. Particles were tracked from release to settlement, and counts were aggregated by source and destination APEI. For those settling outside the APEI network, we identified the nearest APEI and calculated settlement distance, enabling detection of ‘near misses’, instances where larvae settled just beyond protection boundaries. This analysis quantified per-APEI retention, pairwise exchange and dispersal proximity, revealing the strengths and spatial gaps in the modelled APEI network.

#### APEI support score

To quantify each polygon’s contribution to APEI connectivity, we defined an APEI support score that integrates both the volume and diversity of larval exchange. This metric reinforces internal connectivity and reflects whether protected populations can support one another through natural dispersal, promoting long-term persistence. It is based on realized larval dispersal rather than geometric proximity^64^.

The score is calculated as

$${A}_{{\rm{i}}}=\left(S+R\right)\times \left(1+2\times \frac{\min \left(S,R\right)}{S+R}\right)\times \left({N}_{{\rm{s}}}+{N}_{{\rm{r}}}\right),$$

where *S* and *R* are the number of particles sent to and received from APEIs, and *N*_s_ and *N*_r_ are the number of distinct APEIs to which particles were sent or from which they were received, respectively. The middle term represents a role balance factor, scaling from 1 (completely one-sided exchange) to 2 (perfectly reciprocal exchange). Polygons with no exchange (*S* + *R* = 0) were assigned a score of zero. This formulation prioritizes polygons that not only have high exchange but also support bidirectional and broadly distributed connectivity with APEIs. The spatial configuration of grid-cell wise *A* can be seen in Extended Data Fig. 1 (top).

#### CCZ-wide connectivity structure

To assess broad-scale connectivity across the CCZ, we applied a gridded network analysis at 100 km × 100 km resolution (Extended Data Fig. 4), following a species-agnostic ‘everything is everywhere’ framework^23^, and assigned each released particle to a grid cell. Each grid cell was treated as a node, and larval fluxes between cells formed directional, weighted edges. Network metrics—including cell-wise out-degree (source strength), in-degree (sink strength) and betweenness centrality (connector role)—were calculated using the ‘igraph’ package^65^, with self-recruitment excluded from structural metrics to avoid inflating node connectivity (but retained in visualizations and summaries). Community detection was performed on the connectivity network aggregated across all simulated time periods, seasons and PLDs, rather than on individual time slices. This aggregation is intended to identify broad-scale community structure that persists across the range of circulation states considered, rather than transient partitions associated with any single period. Performing detection on the network aggregated across all three PLDs additionally means that the resulting community structure reflects dispersal patterns integrated across the full modelled range of larval durations, rather than being derived from any single dispersal kernel, providing a more stable basis for community identification than PLD-specific networks would. The fast-greedy algorithm^65^ was applied to delineate clusters of high internal and low external connectivity and was selected for its deterministic, parameter-free implementation, which facilitates transparent comparison across baseline and disturbance scenarios without introducing stochasticity or resolution parameters required by alternative methods such as Louvain or Infomap. Community partitions derived from this approach are conditional on the algorithm used and should be interpreted as a comparative framework for identifying broad-scale modular structure rather than definitive or algorithm-independent ecological divisions and should not be interpreted as ecological communities in the traditional biological sense (that is, assemblages of interacting species). Modularity and assortativity were calculated^66,67^ to assess network compartmentalization. Modularity and assortativity values range from −1 to 1, where positive modularity indicates strong internal connectivity within communities and negative or low values suggest weak or diffuse structure. Assortativity reflects whether cells tend to connect with others of similar connectivity, with higher values indicating stronger preference. Community characteristics (size, strength and density) were quantified as the number of cells, total connection weight (that is, the number of particles travelling between cells within the community) and the ratio of realized to possible links, respectively^68^.

### Simulating the impact of mining on CCZ connectivity

To assess how mining may affect CCZ-wide connectivity, we conducted two complementary analyses using a filtered particle dataset in which all particles released from mining contract areas were excluded^2^, representing a scenario in which AMIs no longer function as larval sources.

#### Community reassignment under mining disturbance

To identify transitional zones—cells that shift community affiliation under future disturbance—we re-ran the community detection analysis using the filtered dataset. Each cell’s community identity under baseline and disturbed conditions was compared, and cells with differing assignments were classified as transitional. These zones lie at the boundaries of semi-isolated dispersal communities and represent areas where modelled particle supply from multiple sources contributes to community membership; their sensitivity to mining-driven source loss makes them of particular interest for conservation planning. Cells in the grid were given a binary status (*T*_*i*_) variable (where *i* is the individual cell) set to 1 for transitional zones and 0 otherwise. The identification of transitional zones does not rely on the exact placement of community boundaries; rather, it uses community affiliation as a comparative reference to detect areas prone to network reorganization under disturbance.

#### Larval supply to AMIs under baseline and mining disturbance

To quantify the contribution of APEIs and other CCZ regions to modelled particle replenishment of AMIs, we traced the origin of all particles settling within AMI polygons under both baseline conditions and the mining disturbance scenario. For each origin category—APEI-derived, AMI-origin (encompassing within-AMI retention and inter-AMI exchange) and unprotected non-mining areas—we calculated three complementary metrics: presence-based spatial reach (the proportion of AMI polygons receiving particles above the 1,000-particle threshold), source-normalized flux (the proportion of particles released from APEIs that settled within AMIs) and sink-normalized flux (the proportion of AMI settlers originating from each category). For presence-based spatial reach, we applied a minimum of 1,000 simulated particles aggregated across all simulations as a threshold for meaningful exchange. This value represents an order-of-magnitude filter scaled to the ensemble size of the simulations, retaining exchange with substantial aggregated particle flow while excluding numerically trivial connections that may reflect stochastic dispersal events rather than sustained supply. It is not intended to represent a biological recruitment threshold but to identify AMI sites receiving an level of structurally meaningful particle input. This threshold is applied consistently throughout the connectivity and network optimization analyses (see the ‘Algorithm for APEI network optimization’ section). These metrics were calculated per PLD and aggregated across all simulations. Per-APEI breakdowns of source- and sink-normalized flux are reported in Supplementary Table 1.

For each 100 km × 100 km polygon in the CCZ, we additionally quantified three measures of particle supply to AMIs: (1) the total number of simulated particles originating from that polygon that settled within mining areas, (2) the number of distinct mining areas reached by particles from that polygon and (3) the proportion of particles released from that polygon that settled in at least one mining area. To derive a composite mining replenishment score, *M*_*i*_ (where *i* is the individual cell) (Extended Data Fig. 1, bottom) for each polygon, we applied principal component analysis to the three metrics. Principal component analysis identifies the dominant axis of variation across polygons and combines the metrics into a single score without relying on arbitrary weights. The first principal component (PC1), which captured the majority of variance in the data, was used as the replenishment score. This approach allowed us to objectively rank polygons by their overall importance in replenishing mining areas, integrating total contribution, spatial reach and delivery efficiency into a single continuous metric. The analysis assumed equal habitat suitability across all source cells; habitat heterogeneity, productivity gradients and species distributions could alter the relative importance of individual source areas, and corridor outputs should therefore be evaluated alongside habitat, substrate and carbon flux data as complementary planning layers (Discussion).

### Identifying scenario-stable connectivity hot spots

To identify grid cells that consistently contribute to CCZ-wide connectivity, both now and under future mining scenarios, we developed a scenario-stable connectivity score. This metric integrates two core structural indicators from the gridded network analysis: out-degree (representing source strength) and node betweenness (indicating stepping-stone importance). Each metric was calculated under two scenarios: one using the full particle dataset (including particles released from all areas), and one excluding particles originating within mining contract zones^2^. All four metrics (two per scenario) were min–max normalized independently to account for differences in network structure between scenarios and to ensure equal weighting. As the absolute magnitude and ecological importance of each metric (for example, source strength versus stepping-stone role) are not directly comparable or known, normalization allowed each to contribute proportionately to the composite score^23^. The final scenario-stable score (*S*_*i*_, where *i* is the individual cell) for each cell was calculated as the mean of these normalized values. This approach prioritizes cells that remain important larval contributors and connectors even under potential mining-induced source loss.

### Algorithm for APEI network optimization

To guide the strategic expansion of the APEI network, we developed a spatial prioritization framework to identify high-impact areas for protection, building on the connectivity importance index developed by James et al.^23^. Our approach targets locations that consistently support network function and resilience, offering robust, science-based guidance for spatial planning.

An ICV (Extended Data Fig. 3) for each 100 km × 100 km polygon in the CCZ grid was calculated from the APEI support score (*A*_*i*_), the mining replenishment score (*M*_*i*_) and the scenario-stable connectivity score (*S*_*i*_) with a transitional status indicator (*T*_*i*_) following

$${\mathrm{IC}}{\mathrm{V}}_{i}=\frac{1}{4}\left(\frac{{A}_{i}-{A}_{\min }}{{A}_{\max }-{A}_{\min }}+\frac{{M}_{i}-{M}_{\min }}{{M}_{\max }-{M}_{\min }}+\frac{{S}_{i}-{S}_{\min }}{{S}_{\max }-{S}_{\min }}+{T}_{i}\right)$$

We applied equal a priori weights to the four components of the ICV score, reflecting the multiobjective nature of the prioritization process, where ecological function, management relevance and structural shifts are considered equally important. Assigning equal weight avoids introducing subjective bias towards any one metric and aligns with precautionary, inclusive conservation planning principles. To justify this approach, we assessed pairwise correlations among the components. All correlation coefficients were below 0.5, and the fourth component (reflecting community reassignments) was particularly weakly correlated with the others (*r* < 0.1 in all cases). This confirms that the components contribute complementary information and supports their treatment as distinct and equally weighted in the final score. Equal weighting in this context refers to equal a priori importance of the decision criteria rather than an expectation that each component contributes equally across all locations. Min–max scaling places metrics on a common bounded scale while preserving proportional spatial differences within each component, allowing the prioritization to reflect real variation in connectivity structure. Alternative normalization approaches, such as rank transformations or robust standardization, could reduce sensitivity to distributional differences but would alter the interpretation of metric magnitudes and therefore represent different design choices. The choice of min–max scaling is therefore intended to provide a transparent and interpretable basis for multicriteria prioritization rather than a unique or objective normalization scheme. Alternative weightings could be applied to reflect different management or conservation priorities, for example, upweighting mining replenishment for policy assessments focused on AMI recovery, but exploring such scenarios is beyond the scope of this study; the ICV is intended as a flexible decision-support tool rather than a prescriptive ranking based on fixed value judgements.

Finally, we developed an iterative spatial algorithm to identify the smallest set of additional grid cells needed to connect the existing APEI network via observed larval dispersal paths. First, we ranked all non-APEI polygons by their ICV and incrementally included them until all APEIs were connected through mutual dispersal links, defined as reciprocal exchange exceeding a particle-flow threshold of 1,000 simulated particles aggregated across all simulations, as explained above, ensuring ecological cohesion via high-value areas. We then refined this network using a graph-theoretic optimization approach adapted from Steiner tree methods^69^. Classical Steiner tree formulations minimize geometric or abstract cost distances between terminal nodes; our adaptation operates on the dispersal graph rather than geographic space, reflecting the fact that advective circulation means geographic proximity does not reliably predict exchange strength.

The refinement proceeds in three steps. First, an undirected graph was constructed using only reciprocal exchange links—pairs of polygons exchanging particles in both directions above the 1,000-particle threshold. Edge weights were defined as the inverse of larval flux (1/particles), so that corridors with stronger exchange were assigned a lower cost and preferentially selected by the routing algorithm; a small constant (1 × 10^−6^) was added to prevent division by zero. Second, weighted shortest paths were computed between all pairs of APEI terminal nodes using Dijkstra’s algorithm^70^, identifying the highest-flow route connecting each APEI pair through the available dispersal network. All edges traversed by any of these pairwise shortest paths were collected into a candidate edge set. Third, any polygon appearing on at least one APEI-to-APEI shortest path was retained in the final corridor set; polygons from the phase 1 candidate set that lay on no shortest path were discarded as redundant. This pruning step ensures that every retained corridor cell lies on the strongest available dispersal route between at least one APEI pair.

This union-of-shortest-paths approach is a standard practical approximation to the NP-hard Steiner minimum tree problem; it does not guarantee a globally optimal solution but provides a computationally tractable, flow-optimal result in which all retained corridor cells are biologically justified by their position on high-exchange dispersal pathways between protected areas.

Grid cells identified by the optimization were defined on the same 100 km × 100 km grid as the regional connectivity analysis. Where such cells intersect AMIs, connectivity calculations retained non-mined proportions of cells, while excluding larval contributions originating within AMIs in mining scenarios, thus avoiding imposing a binary disturbance assumption at the grid-cell scale.

### Reporting summary

Further information on research design is available in the Nature Portfolio Reporting Summary linked to this article.

## Supplementary information

Supplementary Information Supplementary Fig. 1. Marginal returns (unique count increase per additional PPS) as a function of PPS. The curve was fitted using a logarithmic model to best represent diminishing returns in unique counts as PPS increases. Vertical orange line marks the point of diminishing returns where the marginal return falls below 1, and the vertical red line denotes the point at which the marginal return is less than 0.1. Policy relevance summary: dispersal corridors enhance modelled near-bed larval connectivity across deep-sea protected area networks. Discusses the policy context for deep-sea mining in the Clarion–Clipperton Zone, the role of larval connectivity in marine spatial planning and protected area network design and key policy-relevant findings from the study. Provides context for decision-makers on how connectivity modelling informs conservation strategy ahead of commercial seabed mining. Also available via Zenodo at https://doi.org/10.5281/zenodo.21413810 (ref. ^35^).

Reporting Summary

Peer Review File

Supplementary Table 1 Per-APEI modelled larval connectivity to AMIs across simulated PLDs. Source-normalized metrics report the proportion of particles released from each APEI that reached AMIs and each APEI’s share of total APEI-to-AMI larval flow. Sink-normalized metrics report each APEI’s contribution as a proportion of all particles settling in AMIs, under baseline conditions (all larval sources active) and under a mining disturbance scenario (AMI self-supply removed). All values are percentages. Aggregated values are pooled across all PLDs and seasons.

## Figures and Tables

**Extended Data Table 1:**
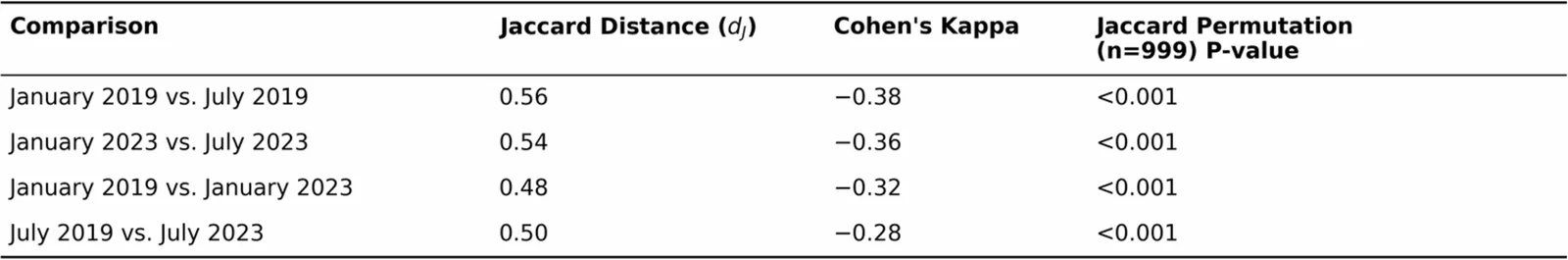
Dissimilarity in larval connectivity between seasons and years across the CCZ

Pairwise comparisons of near-bed larval connectivity between seasons (January vs. July) and years (2019 vs. 2023), quantified by the Jaccard distance (d_J) and Cohen’s κ (reported as the associated effect size; negative values indicate agreement below chance). Statistical significance was assessed with a one-sided permutation test (n = 999 permutations), testing whether observed between-period Jaccard similarity was lower than expected under random label reshuffling. No adjustment was made for multiple comparisons. For all comparisons, P < 0.001.

**Extended Data Table 2:**
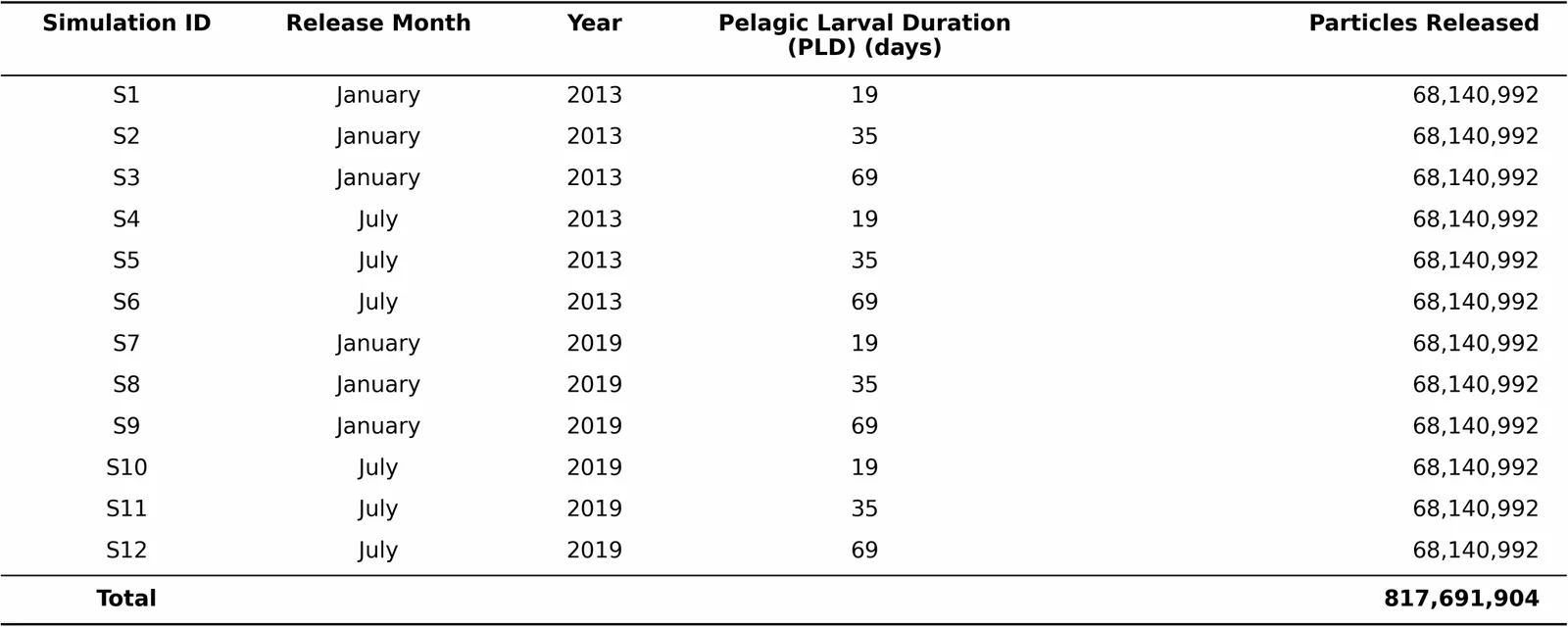
Summary of simulation parameters used in the larval dispersal model

Complete specification of model configuration including: hydrodynamic model (GOFS 3.1 HYCOM), particle release locations and numbers, planktonic larval durations tested (19, 35, 69 days), time periods simulated (January and July 2019, 2023), and computational parameters. Enables full reproducibility and replication of the analysis.

**Extended Data Table 3:**
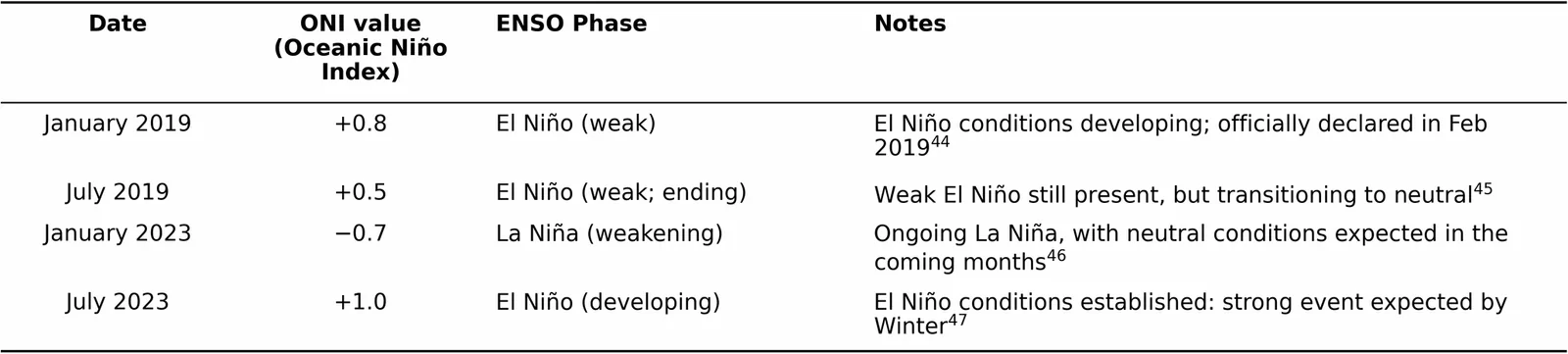
El Niño–Southern Oscillation (ENSO) Phase Classification for particle release dates

ENSO phase classification for January and July 2019 and 2023, based on referenced NOAA reports. Used to assess whether connectivity patterns vary with large-scale oceanographic climate modes.

## Data Availability

Hydrodynamic forcing data were obtained from the GOFS 3.1 HYCOM model and are publicly accessible via the HYCOM Consortium at https://www.hycom.org. The particle-tracking simulations generate very large output datasets that are impractical to archive in a public repository; these outputs, and all derived connectivity data underlying the figures and tables, can be regenerated in full from the publicly available HYCOM input data using the code provided in the ‘Code availability’ section, which reproduces the complete analysis pipeline. A policy relevance statement is available via Zenodo at https://doi.org/10.5281/zenodo.21413810 (ref. ^35^).

## Code availability

All analysis code is publicly available via GitHub at https://github.com/pmlmodelling/ccz-connectivity and via Zenodo at https://doi.org/10.5281/zenodo.21427654 (ref. ^71^). The repository includes full workflow documentation and computational requirements.
