# Supplementary material for: Dispersal corridors enhance modelled near-bed larval connectivity across deep-sea protected area networks
Source: Nat Ecol Evol. 2026 Oct 23;10(11):2129–41. doi: 10.1038/s41559-026-03172-5 (PMC13736681; doi:10.1038/s41559-026-03172-5)
Supplement: Supplementary file 1 [file 41559_2026_3172_MOESM1_ESM.pdf]

nature ecology & evolution

Supplementary information

https://doi.org/10.1038/s41559-026-03172-5

Dispersal corridors enhance modelled near-bed larval connectivity across deep-sea protected area networks

In the format provided by the authors and unedited

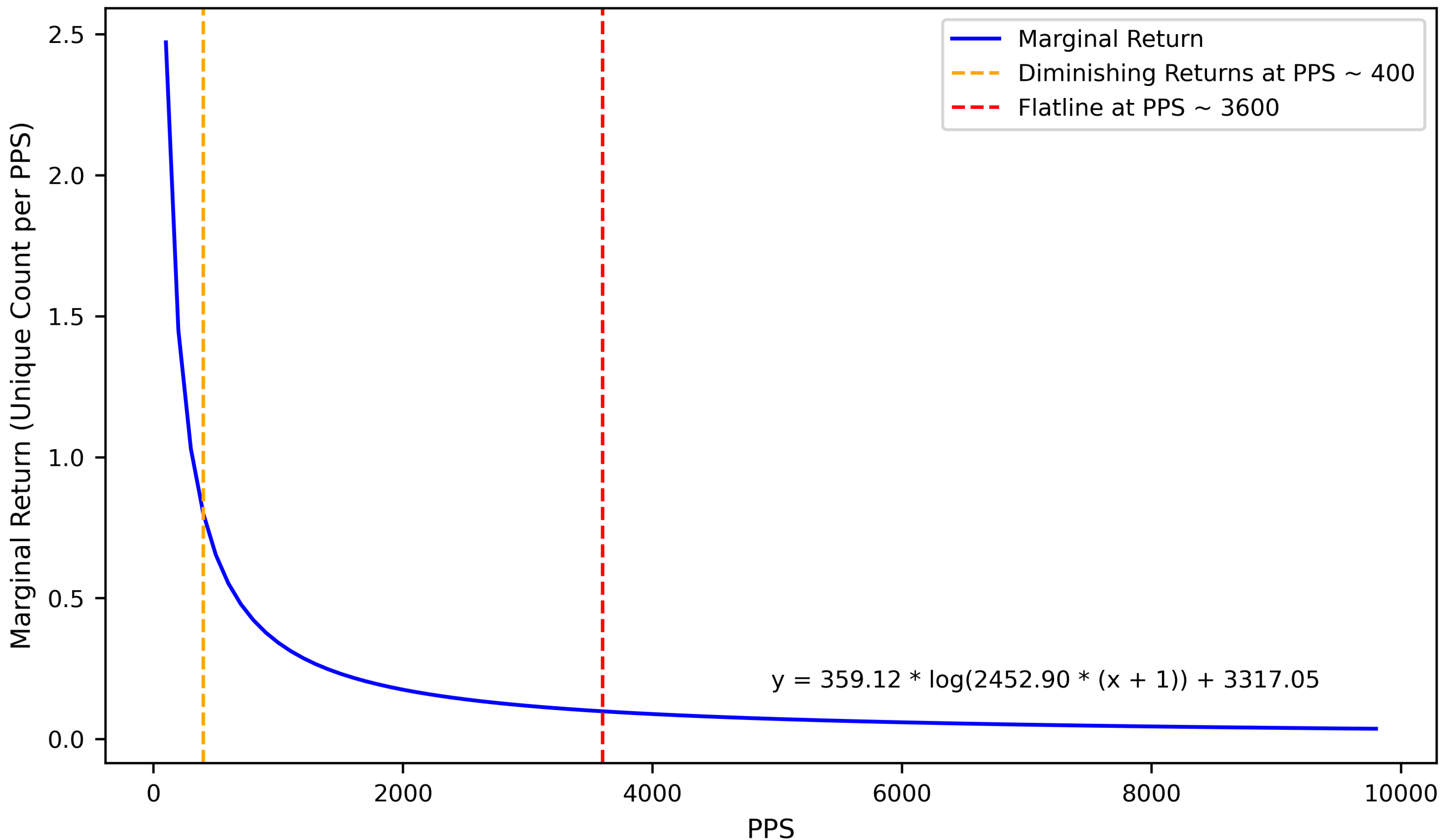

Marginal Return
Diminishing Returns at PPS ~ 400
Flatline at PPS ~ 3600
y = 359.12 * log(2452.90 * (x + 1)) + 3317.05
Marginal Return (Unique Count per PPS)
PPS
0.0
0.5
1.0
1.5
2.0
2.5
0
2000
4000
6000
8000
10000

Dispersal corridors enhance modelled near-bed larval connectivity across deep-sea protected area networks

Policy Relevance Summary

The Current Policy Landscape

The Clarion-Clipperton Zone (CCZ) is the focal region for imminent commercial deep-sea nodule mining. The International Seabed Authority (ISA) has issued exploration contracts for polymetallic nodules across the CCZ to contractors sponsored by states from across the globe, placing over a million km² under exploration licence (ISA, 2025a; ISA, 2025b). Regulatory and commercial pressure toward extraction has intensified markedly: the 2021 Nauru two-year rule trigger created the first legal pathway to proceed without a completed Mining Code (ISA, 2021a); China Minmetals received ISA approval in May 2025 to conduct a CCZ mining trial under exploration regulations (ISA, 2025c); and The Metals Company submitted applications to US regulators in April 2025 to begin commercial extraction outside the ISA framework entirely (AMTI, 2025). The ISA Mining Code remains unfinished after more than a decade of negotiation. The July 2025 Council session concluded without consensus (WRI, 2025), and negotiations were ongoing at the ISA 31st session in March 2026 (ISA, 2026). Once large-scale seabed disturbance begins, the ecological baselines and recovery processes that a protected area network is designed to support will be permanently altered: nodule formation occurs over millions of years, and faunal recovery from physical disturbance is measured in decades to centuries at best (Levin et al., 2016). The spatial configuration of protected areas can be amended, but the ecological context in which they operate cannot be restored. The case for getting the network design right before extraction begins is therefore strong.

How connectivity fits in

In the Regional Environmental Management Plan (REMP for the CCZ, the ISA originally designated nine Areas of Particular Environmental Interest (APEIs) in 2012 to protect regional biodiversity and buffer mining impacts (ISA, 2012; ISA, 2011), expanded to 13 in 2021 (ISA, 2021a). However, connectivity was not a formal design criterion in either the original or the expanded network (ISA, 2011; Smith et al., 2008; Wedding et al., 2013). The 2021 REMP review explicitly acknowledged connectivity as a priority (ISA, 2021b), and the 2019 Friday Harbor workshop had identified genetic connectivity as a key management consideration (ISA, 2019). Multilaterally agreed guidance for MPA networks in the North-East Atlantic requires them to be ecologically coherent – meaning adequate, representative, and connected - as a condition of effective protection (OSPAR, 2003; OSPAR, 2006), and this standard has been reflected in international MPA network design guidance (Gardner et al., 2024). Despite connectivity being an acknowledged aspiration in ISA policy, whether the APEI network actually functions as a connected system had never been formally assessed at region-wide scale. This paper provides that assessment for the first time. Its findings are directly relevant to standards being negotiated in the Mining Code, to the upcoming CCZ REMP review, and to REMPs currently under development for the Mid-Atlantic Ridge, Indian Ocean, and North-West Pacific (ISA, 2024), where equivalent decisions will be made under even greater data scarcity.

A note on uncertainty

The connectivity patterns presented here are modelled estimates conditional on stated physical and biological assumptions and cannot be directly validated against empirical larval exchange data at abyssal depths, where observational coverage is essentially absent. We nevertheless advocate for their use in planning: in a system where biological data are extremely sparse, and management decisions cannot wait for comprehensive empirical evidence, a transparent, physically grounded model is a more defensible basis for area-based management design than no connectivity information at all. The framework is designed to be refined iteratively as empirical data accumulate.

Key findings and policy relevance

Key Finding	Policy Relevance
**Fewer than 0.02% of modelled larvae released from APEIs reach another APEI; only 5 of 156 possible pairwise connections show any exchange**	The network does not meet the ecological coherence standards required by OSPAR (OSPAR, 2003; OSPAR, 2006) and acknowledged as an aspiration in ISA policy (ISA, 2021b). APEIs are functioning as isolated units, not a network
**Median modelled dispersal distances remain below 100 km even at maximum simulated planktonic larval durations (PLDs), far below the 200-800 km gaps between APEIs (Smith et al., 2008)**	The spacing constraints identified at the time of original APEI design (Smith et al., 2008) are confirmed under modelled conditions. Structural improvement requires adding intermediate stepping stones, not redesigning the whole system
**Under the modelled disturbance scenario (where mining source particles are removed), unprotected non-mining areas contribute ~81% of external larval supply to mining zones; current APEIs contribute ~19%**	The ecological resilience the APEI system was designed to deliver (ISA, 2011; ISA, 2012) may depend substantially on areas with no formal long-term protection. Current contract boundaries do not reflect the spatial structure of ecological function
**77% of the highest-connectivity cells fall outside existing APEIs; many lie adjacent to mining contract areas**	Key modelled sources and stepping stones for regional connectivity are largely unprotected. High connectivity-value areas could serve as preservation reference zones (Smith et al., 2008) or inform adaptive management decisions (Wedding et al., 2015)
**CCZ dispersal is structured into four semi-isolated modelled connectivity communities**	The modelled partitioning of the CCZ dispersal landscape is consistent with emerging biogeographic (Simon-Lledó et al., 2023; Simon-Lledó et al., 2025) and genetic evidence of population subdivision (Taboada et al., 2018). The existing network could be augmented by dispersal corridors to enhance modelled connectivity within and between modelled dispersal communities
**Adding strategically placed corridors is modelled to increase inter-APEI connectivity 406-fold and nearly doubles larval contribution from protected areas to areas of mining interest in a commercial mining scenario; ~89% of proposed corridor area does not overlap existing contract areas.**	Meaningful structural improvement is achievable at relatively modest spatial cost (~47% increase in protected area). A scalable, reproducible framework for identifying corridors is now available for CCZ REMP review and future REMPs

Considerations

1. Incorporate connectivity as a measurable criterion in the Mining Code and REMP standards.
The 2021 REMP review acknowledged connectivity as an aspiration (ISA, 2021b) but did not define measurable standards. The Mining Code and future REMPs should move toward quantifiable connectivity criteria, for example, requiring that protected-area configurations be evaluated against best-available connectivity models prior to adoption. Setting standards before exploitation begins is substantially more tractable than retrofitting them post-disturbance.

2. Formally assess the protection status of connectivity-critical unprotected areas.
This paper identifies specific locations outside both APEIs and AMIs that play a disproportionate modelled role in maintaining regional larval supply. Being outside a current exploration contract provides no long-term legal protection. We recommend that the ISA and sponsoring states assess whether existing instruments, including preservation reference zones (Smith et al., 2008) and adaptive management provisions (Wedding et al., 2015), can be applied to areas identified here as connectivity-critical, and that this assessment be incorporated into the next CCZ REMP review.

3. Treat the corridor additions identified here as a priority input to the CCZ REMP review.
The corridors identified by the spatial optimisation provide a concrete, spatially explicit starting point for REMP deliberations. Approximately 89% of their total area does not overlap existing contract zones. They could be evaluated alongside habitat representativity (McQuaid et al., 2020) and biodiversity data (Smith et al., 2021) as complementary planning layers, not as a standalone connectivity solution.

4. Apply this framework to REMPs under development for other regions.
The analytical approach (combining Lagrangian dispersal modelling, network analysis, and the Integrated Connectivity Value metric) was designed for data-limited systems and does not require species-specific biological data to produce planning-relevant outputs. REMPs for the Mid-Atlantic Ridge, Indian Ocean, and North-West Pacific should incorporate connectivity assessment prior to protected-area configuration being finalised (ISA, 2024). The CCZ experience demonstrates the spatial constraints that arise when connectivity is considered only after exploration contracts are in place.

References

AMTI (2025). Between rocks and a hard place: seabed mining in the Pacific. Asia Maritime Transparency Initiative. https://amti.csis.org/between-rocks-and-a-hard-place-seabed-mining-in-the-pacific/. Accessed on 09/02/2026.
Gardner, J.P., Lausche, B., Pittman, S.J. & Metaxas, A. (2024). Marine connectivity conservation: guidance for MPA and MPA network design and management. *Marine Policy*, 167, 106250.
ISA (2011). Environmental management plan for the Clarion-Clipperton Zone. ISBA/17/LTC/7. International Seabed Authority, Kingston.
ISA (2012). Decision of the Council relating to an environmental management plan for the Clarion-Clipperton Zone. ISBA/18/C/22. International Seabed Authority, Kingston.
ISA (2019). Deep CCZ Biodiversity Synthesis Workshop, Friday Harbor Laboratories, Washington. International Seabed Authority, Kingston.
ISA (2021a). Decision of the Council relating to the review of the environmental management plan for the Clarion-Clipperton Zone. ISBA/26/C/58. International Seabed Authority, Kingston.
ISA (2021b). Review of the environmental management plan for the Clarion-Clipperton Zone. ISBA/26/C/43. International Seabed Authority, Kingston.
ISA (2024). Workshop on the development of a regional environmental management plan for the Northwest Pacific. International Seabed Authority. https://isa.org.jm/news/isa-and-japan-host-international-scientific-workshop-for-the-development-of-a-regional-environmental-management-plan-for-the-northwest-pacific/. Accessed on 09/02/2026.
ISA (2025a). Exploration areas. International Seabed Authority. https://isa.org.jm/exploration-contracts/exploration-areas/. Accessed on 09/02/2026.
ISA (2025b). Clarion-Clipperton Fracture Zone. International Seabed Authority. https://isa.org.jm/maps/clarion-clipperton-fracture-zone/. Accessed on 09/02/2026.
ISA (2025c). FAQs for the media. International Seabed Authority. https://www.isa.org.jm/wp-content/uploads/2025/05/FAQs-for-the-Media_28-May-2025.pdf. Accessed on 09/02/2026.
ISA (2026). 31st Session of the International Seabed Authority, Part I, March 2026. https://isa.org.jm/sessions/31st-session-2026/. Accessed on 09/02/2026.
James, M.K., et al. (2025a). The 'everything is everywhere' framework: Holistic network analysis as a marine spatial management tool. *Ecological Informatics*, 80, 103105.
Levin, L.A., et al. (2016). Defining "serious harm" to the marine environment in the context of deep-seabed mining. *Marine Policy*, 74, 245–259.
OSPAR Commission (2003). Guidance on developing an ecologically coherent network of OSPAR Marine Protected Areas. OSPAR, London.
OSPAR Commission (2006). Marine Protected Areas. Reference no. 2006-3. OSPAR, London.
Simon-Lledó, E., et al. (2023). Carbonate compensation depth drives abyssal biogeography in the northeast Pacific. *Nature Ecology & Evolution*, 7(9), 1388–1397.
Simon-Lledó, E., et al. (2025). Marked variability in distance-decay patterns suggests contrasting dispersal ability in abyssal taxa. *Global Ecology and Biogeography*, 34, e13956.
Smith, C.R., et al. (2008). Rationale and recommendations for the establishment of preservation reference areas for nodule mining in the Clarion-Clipperton Zone. ISBA/14/LTC/2. International Seabed Authority, Kingston.
Smith, C.R., Clark, M.R., Goetze, E., Glover, A.G. & Howell, K.L. (2021). Biodiversity, connectivity and ecosystem function across the Clarion-Clipperton Zone: a regional synthesis for an area targeted for nodule mining. *Frontiers in Marine Science*, 8, 797516.
Taboada, S., et al. (2018). Implications of population connectivity studies for the design of marine protected areas in the deep sea: an example of a demosponge from the Clarion-Clipperton Zone. *Molecular Ecology*, 27, 4657–4679.
Wedding, L.M., et al. (2013). From principles to practice: a spatial approach to systematic conservation planning in the deep sea. *Proceedings of the Royal Society B*, 280, 20131684.
Wedding, L.M., et al. (2015). Managing mining of the deep seabed. *Science*, 349, 144–145.
WRI (2025). What we know about deep-sea mining and what we don't. World Resources Institute. https://www.wri.org/insights/deep-sea-mining-explained. Accessed on 09/02/2026.
