# Supplementary material for: Dispersal corridors enhance modelled near-bed larval connectivity across deep-sea protected area networks
Source: Nat Ecol Evol. 2026 Oct 23;10(11):2129–41. doi: 10.1038/s41559-026-03172-5 (PMC13736681; doi:10.1038/s41559-026-03172-5)
Supplement: Supplementary file 3 [file 41559_2026_3172_MOESM3_ESM.pdf]

nature portfolio

Peer Review File

Dispersal corridors enhance modelled near-bed larval connectivity across deep-sea protected area networks

Corresponding Author: Dr Molly James

This manuscript has been previously reviewed at another journal. This document only contains information relating to versions considered at Nature Ecology & Evolution.

Version 0:

Decision Letter:

8th May 2026

*Please ensure you delete the link to your author homepage in this e-mail if you wish to forward it to your co-authors.

Dear Molly,

Your manuscript entitled "Larval dispersal modelling reveals connectivity deficits and priority corridors in a deep-sea protected area network" has now been seen again by 2 of the previous reviewers from your submission to Nature, as well as an additional new reviewer (Reviewer #4) specifically to cover expertise in biophysical modelling and larval dispersal. Their comments are attached (please note that Reviewer #3 from your submission to Nature was unable to re-review).

As you can see, although the original two reviewers were largely satisfied with the revisions, Reviewer #4 raises a number of concerns which will need to be addressed before we can offer publication in Nature Ecology & Evolution. We will therefore need to see your responses to the criticisms raised and to some editorial concerns, along with a revised manuscript, before we can reach a final decision regarding publication.

Specifically, they are concerned that the larval dispersal models are predisposed to low dispersal, and would like to see a much broader exploration of uncertainty in the modelling.

We therefore invite you to revise your manuscript taking into account all reviewer and editor comments. Please highlight all changes in the manuscript text file [OPTIONAL: in Microsoft Word format].

We are committed to providing a fair and constructive peer-review process. Do not hesitate to contact us if there are specific requests from the reviewers that you believe are technically impossible or unlikely to yield a meaningful outcome.

When revising your manuscript:

* Include a “Response to reviewers” document detailing, point-by-point, how you addressed each reviewer comment. If no action was taken to address a point, you must provide a compelling argument. This response will be sent back to the reviewers along with the revised manuscript.

* If you have not done so already please begin to revise your manuscript so that it conforms to our Article format instructions at http://www.nature.com/natecolevol/info/final-submission. Refer also to any guidelines provided in this letter.

* Include a revised version of any required reporting checklist. It will be available to referees (and, potentially, statisticians) to aid in their evaluation if the manuscript goes back for peer review. A revised checklist is essential for re-review of the paper.

* Extended Data Figures - please ensure that any supplementary figures and tables that are crucial to the manuscript’s conclusions are converted into Extended Data figures and tables to increase visibility of these data. Extended Data figures and tables are online-only (present in the online PDF and full-text HTML versions of the paper), peer-reviewed display items that provide essential background to the article but are not included in the main article due to space constraints. A maximum of ten Extended Data display items (figures and tables) is permitted.

Please use the link below to submit your revised manuscript and related files:

Link Redacted

Note: This URL links to your confidential home page and associated information about manuscripts you may have submitted, or that you are reviewing for us. If you wish to forward this email to co-authors, please delete the link to your homepage.

We hope to receive your revised manuscript within four to eight weeks. If you cannot send it within this time, please let us know. We will be happy to consider your revision so long as nothing similar has been accepted for publication at Nature Ecology & Evolution or published elsewhere.

Nature Ecology & Evolution is committed to improving transparency in authorship. As part of our efforts in this direction, we are now requesting that all authors identified as 'corresponding author' on published papers create and link their Open Researcher and Contributor Identifier (ORCID) with their account on the Manuscript Tracking System (MTS), prior to acceptance. ORCID helps the scientific community achieve unambiguous attribution of all scholarly contributions. You can create and link your ORCID from the home page of the MTS by clicking on 'Modify my Springer Nature account'. For more information please visit please visit www.springernature.com/orcid.

Please do not hesitate to contact me if you have any questions or would like to discuss these revisions further.

We look forward to seeing the revised manuscript and thank you for the opportunity to review your work.

[redacted]

Reviewers' comments:

Reviewer #1 (Remarks to the Author):

The authors have done a nice job revising the paper to address my comments (Reviewer # 1). This mainly has consisted of rewriting rather than conducting new/additional analyses. This includes (1) better discussion of the rationale and limitations of their assumptions, including the "everything is everywhere assumption," and (2) presenting results as hypotheses based on the outputs of modeling (rather than as quantitative predictions of natural patterns). They have also moderated the overly strong statements concerning their results, have highlighted the importance of maintaining the current large APEIs while adding connectivity corridors, and have better represented the APEI literature. They also discuss the point that incorporating spatially variable larval production driven by POC flux, or incorporating habitat suitability, could redistribute the relative importance of sources and stepping-stone regions in dispersal corridors. Finally, they have suggested data collection that could improve the modeling results in the future.

Nonetheless, I do recommend some minor changes.

Suggested title change: Larval-dispersal modelling suggests low connectivity across a deep-sea protected-area network can be enhanced by dispersal corridors

Fourth sentence of abstract, change to: "Under physically driven near-bed dispersal, and reasonable pelagic larval dispersal times of 19-69 d, simulated exchange among APEIs is extremely limited: fewer than 0.02% of particles reach another APEI, with inter-APEI gaps exceeding modelled dispersal capacity." I.e., add the PLD range used in the study. This is fundamental to interpreting the results.

First sentence of Results, change to: "Simulations incorporated multiple seasons, years, and reasonable planktonic larval durations (PLDs) ranging from 19 – 69 d (see Methods) to capture environmental and biological variability relevant to management-scale planning."
First sentence of second paragraph of Results, change to: "Simulated exchange among APEIs was extremely limited for PLDs up to 69 d (Figure 1)."

I think the Discussion should better capture the fact that the modeling likely does not reflect the full potential range of physical variability in the region (as pointed out in the Methods). Thus, I suggest that in the second to last paragraph of the Discussion, after "Connectivity patterns are aggregated across four simulated time periods spanning seasonal and interannual variability, and across three PLDs representing a plausible range for deep-sea taxa; this provides an integrated representation across simulated conditions rather than a formal uncertainty analysis.", the authors insert something like: "It should be noted, however, that these time periods do not encompass the full range of ENSO variability, nor longer-term decadal circulation patterns; results should be interpreted as integrating the circulation states of the four time intervals modeled rather than as long-term climatological averages."

Otherwise, the discussion is well written but has many long paragraphs that could easily be broken up to enhance readability.

The Policy Relevance Summary is a substantial strength. However, I still think that the importance of dispersal Community 2 is overstated in Key Finding 5: “CCZ dispersal is structured into four semi-isolated modelled connectivity communities; one (Community 2: the smallest and most internally connected) has no APEI coverage.” Community 2 may well not be the smallest since it clearly extends beyond the modeled area, and it is so far from any AMI that it is effectively protected from mining and an APEI for mining is not warranted (as now indicated in the main body of the paper). Thus, I suggest deleting the words “one (Community 2: the smallest and most internally connected) has no APEI coverage.” from the end of this paragraph.

Also, for Key Finding Five, the Policy Relevance statement (“The existing network does not reflect the modelled partitioning of the CCZ dispersal landscape, consistent with emerging biogeographic (Simon-Lledó et al., 2023; Simon-Lledó et al., 2025) and genetic evidence of population subdivision (Taboada et al., 2018).”) should be moderated. In fact, all the Communities with AMI have 3 – 6 broadly distributed APEIs. The main issue here is connectivity, not representativity. Change this statement to: “The existing network could be augmented by dispersal corridors to enhance modelled connectivity within and between modelled dispersal communities.”

Overall, the authors have revised the paper to adequately address most of my comments/concerns. However, Reviewers 2 and 3 have raised additional concerns which I cannot address. In particular, Reviewer 2 raises points regarding modeling assumptions, classification of connectivity, calculation of Integrated Connectivity Value (ICV) scores, and other interpretations of results; I think Reviewer 2 (or an editor competent in these areas) needs to judge whether these issues have been addressed. If the concerns of Reviewers 2 and 3 have been adequately addressed, I would very much like to see the paper published. The paper is very timely, well conceived and written, and should be of broad interest to scientists, environmental managers, and policy makers. It has the potential to substantially improve environmental protection in the face of nodule mining in the CCZ by facilitating implementation/protection of dispersal corridors in a region (the CCZ) targeted for deep-sea mining in the near future, and could provide a conceptual framework for integrating connectivity and dispersal corridors into protected-area design in other deep-sea regions targeted for mining.

Reviewer #2 (Remarks to the Author):

The authors have satisfactorily addressed my comments from the last time I reviewed this paper, and I think it is much stronger now and suitable for publication. I have some minor comments below, but do not need to review this paper again. I congratulate the authors on an important and interesing analysis.

line 7: fewer than 15 particles each is hard to frame in a paper where the methods come after the results. A reasonable reader can guess that you released many particles so 15 is a relatively small amount, but we don’t know. Suggest saying 15 particles (X% of those released) or something like that

194: I think elsewhere in the paper the authors refer to this as “mining disturbance” scenario (mining extirpation maybe?) I would suggest consistent usage of that full phrase rather than just “mining”, which implies that “mining” intrinsically equals no self replenishment. It is a possibility and as a worst-case scenario worth exploring here, but not sure fair to imply that mining = automatic localized extirpation.

319: Not sure I totally buy the “it’s intrinsically better to not include poorly understood processes” implicit here. Not including them in this case is the same as saying the vertical movement does not occur; it does not remove vertical movement from the equation, it simply makes one specific assumption about it. That this assumption is inherently conservative is not obvious to me; I dont’ know enough about abyssal currents to know if currents are stronger near or far from the seabed, if there are layers with different current directions. It seems possible to me that connectivity might be even lower if vertical movement is meaningful and currents are slower higher in the water column, or head in different directions than those on the seabed. All to say, I do not think the authors need to include vertical movement, but would suggest a bit more nuance in this and other statements that seem to imply that it is not just necessary (we don’t have the data/models to do fine scale) but intrinsically better to use this particular set of simplifying assumptions, rather than say explicitly parameterizing and integrating over uncertain states.

657: “We applied equal weights to the four components of the ICV score to ensure that no single criterion dominates the outcome. “ Same critique as last time with this wording; this equation has no guarantee that no single criterion dominates the outcomes. The effective weighting is a function of the actual skew in the distribution of each of the components, so while this equation appears “neutral” in practice it could be highly skewed towards only one metric. I am OK with the authors leaving the equation as is, but simply changing the wording to not create the impression that this equation enforces equal contribution to the outcome, rather than simply allowing it. The phrasing here also follows similar tendencies regarding model structure that seems to imply that there is something intrinsically “best” about the supplied approach, as opposed to it being a choice that was made with tradeoffs like any other. The choice of what weighting scheme to use is just as subjective as the choice of specific weighting functions. Using a rank transformation would also not require “explicit” weightings. I am not saying that the authors need to change anything here, but would urge more nuanced language here and throughout that does not seem to imply that the selected approach is in some way more or less “objective” than some other approach. All modeling choices are subjective to some degree, and there are plenty of other weighting schemes that are just as “objective” as this one. Also, as before, the diagnostic here in terms of risk of the “posterior” weighting being skewed towards one

component would be the skew of the distributions of each component, not the correlations per se (though this is an useful piece of information as well).

Many thanks for providing the code, it was not made available to me as a reviewer in the last round, apologies if that was a journal issue rather than a choice by the authors.

Reviewer #2 (Remarks on code availability):

I have reviewed the repository and confirmed that it contains a self-reported reproducible workflow for the products produced here. I have not actually examined the specific scripts, or attempted to reproduce it myself. The README has very clear instructions for reproducing the results.

Reviewer #4 (Remarks to the Author):

This is an ambitious, timely, and policy-relevant manuscript, and the integration of abyssal particle tracking with network optimisation is potentially very valuable for CCZ spatial planning. However, my methodological concerns are substantial and currently limit confidence in the strength of the manuscript's central conclusions.

My principal concern is that the manuscript's inference of severe connectivity deficits is derived from a parameter space that appears strongly predisposed to minimise dispersal. In particular, the combination of relatively short planktonic larval durations and strict benthic boundary-layer transport is likely to bias estimates toward low exchange among APEIs. While the authors present this as a conservative baseline, the biological justification is not yet sufficiently convincing. Deep-sea taxa span a wide diversity of dispersal strategies, including direct developers, long-duration larvae, taxa with ontogenetic vertical shifts, deep-sea fishes whose larvae occupy shallower depths than adults, and vent-associated taxa that must access faster off-bottom currents to bridge habitat gaps of hundreds to thousands of kilometres. By not exploring this broader biologically defensible parameter space, the manuscript risks materially underestimating both inter-APEI connectivity and the contribution of protected areas to replenishment.

This issue is fundamentally one of uncertainty treatment. The manuscript would be much stronger if it explicitly explored parameter uncertainty, rather than relying on a narrow set of representative assumptions. The same logic used to examine sensitivity to PLD should be extended to vertical positioning in the water column and to a wider PLD range. Other deep-sea dispersal studies have considered durations extending to many months or even approximately one year for some taxa. Without testing whether the connectivity matrix changes under these plausible alternatives, the current conclusions remain too conditional to support strong claims about the structural incoherence of the APEI network.

I also encourage reconsideration of how connectivity importance is quantified. The ≥1,000 particle threshold is difficult to interpret ecologically in isolation. For revision, it would be more informative to express source importance as the proportional contribution of APEIs relative to all competing sources for a given destination cell. Likewise, the justification for selecting the "top 10%" of high-connectivity cells requires clearer rationale or sensitivity testing.

The clustering and community-detection analyses also need clearer methodological support. The manuscript should explain more directly what features of the hydrodynamic field generate the observed partitions despite uniform particle release, whether these partitions align with known CCZ biogeographic provinces, and how robust boundary assignments are to changes in dispersal kernels. Cells near community edges are unlikely to have equally certain cluster membership, and this uncertainty is likely central to the disturbance scenario.

Finally, the disturbance scenario likely remains conservative because it treats mining effects primarily as source removal. Sediment plumes extending beyond AMIs could increase mortality for near-bed larvae and further reduce both local replenishment and regional stepping-stone exchange. This possibility needs clearer acknowledgement when discussing resilience and recovery potential.
In summary, the manuscript is promising, but the current methodological framework is too narrowly parameterised to fully support the strength of the headline conclusions. Broader biologically realistic sensitivity analyses are needed before the claims regarding CCZ protected-area network coherence can be considered robust.

Detailed feedback list aligned to likely manuscript locations

p4, Results opening: The 'everything is everywhere' / uniform release assumption needs stronger justification given empirical evidence that abyssal larval assemblages are spatially patchy (e.g., Kersten et al. 2019).

p6, Table 1 / AMI replenishment: Replace or complement the ≥1,000 particle threshold with proportional source contribution metrics. Absolute counts alone may obscure biologically meaningful relative supply.

p8, Figure 2: Clarify whether dominance of unprotected sources is partly a geometric consequence of proximity to AMIs under limited dispersal assumptions.

p10, Figure 3: Clarify what network properties define communities and what hydrodynamic barriers create these partitions despite uniform release.

p12, Figure 4: Justify the choice of ‘top 10%’ high-connectivity cells and report the fraction of prioritised area outside AMIs and APEIs more explicitly.

p16-18, Discussion constraints: Current assumptions are not yet sufficiently well supported. Explicit uncertainty analyses across PLD and vertical position would be more robust than relying on a narrow assumed range.

p18, Discussion: Acknowledge that short PLDs and strict BBL confinement may systematically understate connectivity for taxa with longer-duration or off-bottom larvae.

p20-22, Methods hydrodynamics and particle tracker: The rationale for using only January and July in two years needs evidence that these months are representative of broader circulation states.

p21-22, Vertical depth assumption: The argument for constraining particles to bottom flow is not convincing for all taxa. Explore shallower larval depths as a formal sensitivity analysis.

p25-26, Accounting for variation / PLD: Quantify how the connectivity matrix changes as a function of PLD rather than only changes in dispersal-distance kernels.

p27-28, Community detection: Provide robustness checks or confidence measures for community assignment, especially for boundary cells where membership may be uncertain.

p29, Mining scenario: Expand disturbance scenarios to discuss potential mortality effects of sediment plumes on near-bed larvae, not only source removal.

********************END********************

Version 1:

Decision Letter:

26th June 2026

Dear Dr. James,

Thank you for submitting your revised manuscript "Near-bed larval-dispersal modelling suggests low connectivity across a deep-sea protected-area network can be enhanced by dispersal corridors" (NATECOLEVOL-26031276A). It has now been seen again by the original reviewers and their comments are below. The reviewers find that the paper has improved in revision, and therefore we'll be happy in principle to publish it in Nature Ecology & Evolution, pending minor revisions to comply with our editorial and formatting guidelines.

If the current version of your manuscript is in a PDF format, please email us a copy of the file in an editable format (Microsoft Word or LaTex)-- we can not proceed with PDFs at this stage.

If you have not done so already, please ensure that you also email us a completed copy of the Reporting summary :

Reporting summary: https://www.nature.com/documents/nr-reporting-summary.pdf

We are now performing detailed checks on your paper and will send you a checklist detailing our editorial and formatting requirements in a couple of weeks. Please do not upload the final materials and make any revisions until you receive this additional information from us.

Thank you again for your interest in Nature Ecology & Evolution. Please do not hesitate to contact me if you have any questions.

[redacted]

Reviewer #1 (Remarks to the Author):

I believe the authors have adequately addressed my relatively minor concerns and suggestions. If the other reviewers are satisfied (in particular Reviewer # 4), I recommend publication.

Reviewer #4 (Remarks to the Author):

I thank the authors for providing detailed and defensible arguments in response to my queries and those of the other reviewers. I am happy with the softening of the language and for making the assumptions clearer and providing additional justification in the methods and testing of parameter values in the extend data. I have no further suggested changes.

Open Access This Peer Review File is licensed under a Creative Commons Attribution 4.0 International License, which permits use, sharing, adaptation, distribution and reproduction in any medium or format, as long as you give appropriate credit to the original author(s) and the source, provide a link to the Creative Commons license, and indicate if changes were made.
In cases where reviewers are anonymous, credit should be given to 'Anonymous Referee' and the source.
The images or other third party material in this Peer Review File are included in the article's Creative Commons license, unless indicated otherwise in a credit line to the material. If material is not included in the article's Creative Commons license and your intended use is not permitted by statutory regulation or exceeds the permitted use, you will need to obtain permission directly from the copyright holder.
To view a copy of this license, visit https://creativecommons.org/licenses/by/4.0/

Response to Reviewer 1

We thank Reviewer 1 for their positive assessment of the revised manuscript and for the constructive minor suggestions. All recommended changes have been implemented as described below.

Summary of changes: The title has been revised, the PLD range has been added to the abstract and key Results sentences, the suggested ENSO caveat has been inserted in the Discussion, and Key Finding 5 of the Policy Relevance Summary has been revised as recommended. Long Discussion paragraphs have also been broken up to improve readability.

Detailed changes:

Comment: Suggested title change: *Larval-dispersal modelling suggests low connectivity across a deep-sea protected-area network can be enhanced by dispersal corridors.*

Response: We have revised the title largely as suggested. We have additionally incorporated "Near-bed" as a qualifier - "Near-bed larval-dispersal modelling suggests low connectivity across a deep-sea protected-area network can be enhanced by dispersal corridors" - to make the near-bed transport assumption explicit from the outset, addressing a related concern raised by Reviewer 4.

Comment: Fourth sentence of abstract, change to include the PLD range (19-69 d).

Response: Done. The abstract now reads: "Under physically driven near-bed dispersal, and reasonable pelagic larval dispersal times of 19-69 d, simulated exchange among APEIs is extremely limited: fewer than 0.02% of particles reach another APEI, with inter-APEI gaps exceeding modelled dispersal capacity."

Comment: First sentence of Results, change to include "reasonable planktonic larval durations (PLDs) ranging from 19-69 d."

Response: Done. The Results now opens: "Simulations incorporated multiple seasons, years, and reasonable planktonic larval durations (PLDs) ranging from 19-69d (see Methods) to capture environmental and biological variability relevant to management-scale planning."

Comment: First sentence of second paragraph of Results, change to "Simulated exchange among APEIs was extremely limited for PLDs up to 69 d (Figure 1)."

Response: Done. The sentence now reads: "Simulated exchange among APEIs was extremely limited for PLDs up to 69d (Figure 1)."

Comment: Insert a sentence in the second to last paragraph of the Discussion noting that the four time periods do not encompass the full range of ENSO variability or longer-term decadal circulation patterns.

Response: Done. The following sentence has been inserted after "this provides an integrated representation across simulated conditions rather than a formal uncertainty analysis": "It

should be noted, however, that these time periods do not encompass the full range of ENSO variability, nor longer-term decadal circulation patterns; results should be interpreted as integrating the circulation states of the four time intervals modelled rather than as long-term climatological averages."

Comment: The discussion has many long paragraphs that could easily be broken up to enhance readability.

Response: We have broken up several long Discussion paragraphs to improve readability.

Comment: Key Finding 5 of the Policy Relevance Summary - delete "one (Community 2: the smallest and most internally connected) has no APEI coverage."

Response: Done. This parenthetical has been removed.

Comment: Key Finding 5 policy relevance statement should be moderated from a representativity claim to a connectivity claim. Change to: "The existing network could be augmented by dispersal corridors to enhance modelled connectivity within and between modelled dispersal communities."

Response: Done. The policy relevance statement has been revised as suggested.

Response to Reviewer 2

We thank Reviewer 2 for their positive assessment and for confirming the manuscript is suitable for publication. We have addressed all remaining minor comments as described below.

Summary of changes: The percentage of released particles has been added alongside the absolute count for the two weakest APEI-to-APEI links; "mining disturbance scenario" terminology has been applied consistently throughout; the vertical depth assumption section has been substantially revised to address the concern about implicit framing of near-bed confinement as intrinsically preferable, including new empirical analysis; and the ICV equal weighting sentence has been revised.

Detailed changes:

Comment (line 7): Fewer than 15 particles each is hard to frame without knowing total release numbers. Suggest expressing as a percentage of those released.

Response: Done. The text now reads: "fewer than 15 particles each: <0.00014% of particles released from those APEIs."

Comment (line 194): Consistent use of "mining disturbance scenario" rather than just "mining" is recommended, to avoid implying mining intrinsically equals no self-replenishment.

Response: Done. "Mining disturbance scenario" is now used consistently throughout the manuscript wherever this scenario is referred to, including in table headers, figure captions, Results, and Methods.

Comment (line 319): The framing implies near-bed confinement is intrinsically better than including poorly understood processes, rather than being a specific modelling choice with its own assumptions and consequences. More nuanced language is requested.

Response: We agree with this critique and have revised both the Methods and the relevant Discussion paragraph. In the Methods, the revised text explicitly acknowledges that constraining particles to near-bed flow "is a specific assumption about larval vertical position rather than an absence of assumption." To assess the physical consequences of this choice empirically rather than assertively, we have added new analysis of HYCOM velocity profiles across the CCZ, sampling every three days across all four simulation windows (92 instantaneous snapshots), comparing terrain-following near-bed velocities against full water column profiles at all 40 HYCOM depth levels (Extended Data Fig Xa). This demonstrates that near-bed speeds (0.033 ± 0.004 m/s) are among the lowest in the water column, with speeds throughout the 2,500–5,000 m depth range essentially equivalent to or marginally slower than near-bed (ratio 0.93–1.03×). We have further added a reachability analysis using the only available empirical deep-sea larval swimming speed measurements (0.2–0.6 mm s^{-1}), quantifying depths reachable within the modelled PLD range and cross-referencing against the HYCOM speed and direction profiles (Extended Data Figs Xb, Xc). The revised text makes clear that near-bed confinement is demonstrably rather than assertedly conservative for taxa swimming at or below the lower bound of measured speeds, while

explicitly acknowledging that taxa with sustained upward swimming capacity and longer PLDs could access faster currents in different directions, representing dispersal pathways not captured by this framework.

In the Discussion, the paragraph previously reading "Vertical behaviour was excluded because parameterising processes that are empirically unconstrained risks compounding existing model error... This represents a conservative physical baseline aimed to avoid overstating connectivity through speculative parameterisation" has been revised to explicitly frame near-bed confinement as a specific assumption rather than an intrinsically preferable choice, retaining the rationale that introducing poorly constrained behavioural parameters risks compounding rather than reducing model uncertainty^33^, and pointing the reader to the Methods for the empirical basis of the conservative characterisation. Reported dispersal distances are stated to be conditional on the near-bed depth constraint rather than upper bounds on dispersal potential for all CCZ taxa.

Comment (line 657): The wording "to ensure that no single criterion dominates the outcome" implies the equation guarantees equal contribution, which it does not. More nuanced language is requested.

Response: Done. The opening sentence has been revised to: "We applied equal a priori weights to the four components of the ICV score, reflecting the multi-objective nature of the prioritisation process, where ecological function, management relevance, and structural shifts are considered equally important." The existing clarification sentence later in the paragraph "Equal weighting in this context refers to equal a priori importance of the decision criteria rather than an expectation that each component contributes equally across all locations" has been retained.

Response to Reviewer 4

We thank Reviewer 4 for their detailed and constructive engagement with the manuscript. Before addressing individual points, we would like to briefly contextualise our approach, as we feel this underpins several of the concerns raised.

This study is explicitly designed as a species-agnostic regional planning framework for a system characterised by extreme data scarcity. Its purpose is to provide a physically grounded, reproducible baseline assessment of structural connectivity across the CCZ APEI network under a transparent and biologically defensible set of assumptions, to inform spatial planning decisions that must be made ahead of commercial mining, and to be refined iteratively as species-specific empirical data accumulate. It is not intended as an exhaustive characterisation of connectivity for all CCZ taxa, nor as a resolution of all sources of uncertainty in deep-sea dispersal modelling, both of which would require empirical data that do not currently exist for CCZ nodule fauna.

Reviewer 4's principal concerns relate to the PLD range, near-bed confinement, and the absence of formal sensitivity analyses across these parameters. In response, the most substantive revisions in this round are: substantially expanded justification of the PLD range in the Methods, grounded explicitly in the Hilário et al. (2015) cross-taxon synthesis and its specific recommendation for MPA planning; and new empirical analysis of HYCOM vertical velocity profiles, larval swimming speed reachability, and flow direction across depth levels, providing an empirical rather than solely assertive basis for the near-bed confinement assumption. Full details of these revisions are provided in the point-by-point responses below.

On the absence of formal sensitivity analyses across PLD and vertical positioning: these omissions are intentional rather than oversights, and the rationale for each is provided in full in the point-by-point responses below. Sensitivity analyses are only informative when the parameter space being explored is empirically bounded: without empirical constraints on deep-sea larval swimming speeds, vertical positioning behaviour, physiological tolerances, or taxon-specific PLDs for CCZ nodule fauna, any sensitivity testing would amount to exploring arbitrary parameter combinations rather than biologically meaningful alternatives, and would risk producing outputs driven by assumption rather than evidence.

Critically, all of the parameters Reviewer 4 suggests exploring - longer PLDs, shallower larval depths, and access to faster mid-water currents - would, if anything, increase connectivity relative to the modelled baseline. Taxa with these characteristics have greater dispersal potential and are therefore less vulnerable to the structural connectivity deficits identified here. The organisms of greatest conservation concern in the context of mining-driven connectivity loss are precisely those with limited dispersal capacity - dependent on near-bed passive transport within biologically grounded PLD ranges - for whom the current framework is most directly applicable. A planning framework designed around taxa with exceptional dispersal strategies would systematically under-protect the majority of CCZ nodule fauna. Designing conservation measures that are robust for organisms with limited dispersal capacity ensures they are also sufficient for more broadly dispersing taxa, but not vice versa. The precautionary logic of the near-bed passive transport baseline is therefore not a limitation of the framework but a deliberate and defensible design choice for conservation planning under data scarcity.

We hope that the revisions made in this round, together with the point-by-point responses provided above, adequately address Reviewer 4's concerns. We believe the manuscript is stronger for this engagement and thank Reviewer 4 for the rigour and depth of their review.

Detailed comments:

Comment: The manuscript's inference of severe connectivity deficits is derived from a parameter space that appears strongly predisposed to minimise dispersal. The combination of relatively short PLDs and strict benthic boundary-layer transport is likely to bias estimates toward low exchange among APEIs. The biological justification for this parameter space is not yet sufficiently convincing, and by not exploring the broader biologically defensible parameter space the manuscript risks materially underestimating inter-APEI connectivity. This issue is fundamentally one of uncertainty treatment - the same logic used to examine sensitivity to PLD should be extended to vertical positioning in the water column and to a wider PLD range. Other deep-sea dispersal studies have considered durations extending to many months or even approximately one year for some taxa. Without testing whether the connectivity matrix changes under these plausible alternatives, the current conclusions remain too conditional to support strong claims about the structural incoherence of the APEI network.

Response: We thank Reviewer 4 for this substantive concern and have made extensive revisions to both the PLD justification and the vertical depth assumption in response. We would also note that the manuscript has substantially moderated its claims in response to the previous round of review. Results are explicitly framed as conditional outputs under a consistent physical framework rather than as predictions of natural patterns, the title has been revised from "reveals" to "suggests", and conclusions are presented as hypotheses based on modelling outputs rather than as quantitative predictions. We therefore consider the strength of the claims made to be appropriate given the modelling framework employed.

On PLDs: the Methods have been substantially revised to ground the 69-day ceiling explicitly in the Hilário et al. (2015) cross-taxon synthesis and its specific recommendation for MPA planning in the deep sea. Hilário et al. recommend that MPA planning should use a PLD that guarantees a minimum dispersal distance for a wide range of species, and their analysis finds that 69 days satisfies this criterion for 75% of eurybathic and deep-sea species. The three modelled durations (19, 35, and 69 days) correspond explicitly to the lower interquartile boundary, median, and upper interquartile boundary of this pooled distribution. We acknowledge that longer PLDs have been recorded for some deep-sea taxa, and the revised text states this explicitly. However, extending beyond the 75th percentile of the Hilário et al. distribution would risk skewing a species-agnostic planning framework toward the dispersal capacity of a small minority of potentially atypical long-ranging taxa, producing outputs unrepresentative of the broader community. Empirical PLD data for CCZ nodule fauna specifically are absent, and this absence reinforces rather than undermines the rationale for a cross-taxon approach: in the absence of taxon-specific data, the Hilário et al. synthesis provides a more defensible and representative basis for regional planning than any single-taxon parameterisation would. The conclusions of the study are explicitly stated to be conditional on the modelled framework and do not claim to represent the full biological diversity of dispersal strategies in the CCZ.

On vertical depth: Reviewer 4 raises a legitimate concern that near-bed confinement may not be appropriate for all CCZ taxa, and that shallower larval depths should be explored as a formal sensitivity analysis. We have added new empirical analysis to address this directly rather than relying solely on assertion. HYCOM velocity profiles were examined across the CCZ every three days across all four simulation windows (92 instantaneous snapshots), comparing terrain-following near-bed velocities against full water column profiles at all 40

HYCOM depth levels (Extended Data Fig Xa). This demonstrates that near-bed speeds (0.033 ± 0.004 m/s) are among the lowest in the water column, with speeds throughout the 2,500–5,000 m depth range essentially equivalent to or marginally slower than near-bed (ratio 0.93–1.03×). A reachability analysis using the only available empirical deep-sea larval swimming speed measurements (0.2–0.6 mm s^{-1} from vent polychaetes and cold-water coral larvae) quantifies depths reachable within the modelled PLD range and cross-references against HYCOM speed and direction profiles (Extended Data Figs Xb, Xc). At the lower swimming speed bound, larvae from CCZ seabed depths would remain within the depth range where current speeds are essentially equivalent to near-bed (ratio 0.93–0.95×), confirming near-bed transport as a reasonable proxy for these taxa. At the upper bound, larvae could theoretically reach depths where currents are 1.3–2.8× faster and directional offsets could reach 90–137° - but it is critical to note that these reachability estimates assume continuous sustained unidirectional upward swimming throughout the full PLD, which is biologically unrealistic for any larva. In practice, larvae alternate between swimming bouts, passive sinking, and feeding, meaning they would either reach shallower depths than the theoretical maxima or take substantially longer to reach any given depth - in either case reducing the time spent in faster mid-water currents and limiting any dispersal advantage of vertical migration. Additionally, ascending larvae would encounter progressively warmer temperatures and lower pressures, conditions of unknown tolerance for CCZ nodule fauna and a potential physiological barrier independent of swimming capacity.

We would also note a practical constraint on formal sensitivity testing at shallower depths: HYCOM velocity outputs at abyssal depths span 1,000 m vertical intervals, meaning off-bottom transport cannot be parameterised at ecologically meaningful depth resolution even if sufficient biological data existed to justify it. Beyond this, parameterising vertical behaviour at shallower depths would require assumptions about swimming speed, duty cycle, physiological tolerances, and depth-dependent behaviour: none of which are empirically constrained for CCZ nodule fauna. Introducing these unconstrained parameters risks compounding rather than reducing model uncertainty. Reported dispersal distances are explicitly stated to be conditional on the near-bed depth constraint and do not represent upper bounds on dispersal potential for all CCZ taxa, and characterising connectivity for taxa with exceptional strategies, whether longer PLDs, active vertical migration, or both, requires species-specific data and targeted modelling beyond the scope of this species-agnostic regional planning framework.

We would also note that Reviewer 4 cites deep-sea fishes and vent-associated taxa as examples of organisms with greater dispersal potential. We agree these taxa exist and may have substantially greater dispersal potential than modelled here. However, the paper explicitly addresses CCZ nodule fauna - the taxa directly relevant to nodule mining impacts - for which available field observations suggest BBL concentration rather than water column ascent, and for which no empirical swimming speed or PLD data exist. The framework is designed as a reproducible baseline to be refined as species-specific empirical data accumulate.

Comment: The ≥1,000 particle threshold is difficult to interpret ecologically in isolation. It would be more informative to express source importance as the proportional contribution of APEIs relative to all competing sources for a given destination cell. The justification for selecting the "top 10%" of high-connectivity cells requires clearer rationale or sensitivity testing.

Response: On the ≥1,000 particle threshold: we agree that absolute particle counts alone are difficult to interpret ecologically in isolation, and the submitted manuscript already reported two complementary proportional metrics alongside the presence-based threshold metric in Table 1. Source-normalised flux reports the proportion of APEI-released particles reaching AMIs, and sink-normalised flux partitions all AMI larval settlers by origin category - APEI-origin, AMI-origin, and unprotected non-mining areas - across all simulated PLDs, with per-APEI breakdowns in Extended Data Table 1. These aggregate sink-normalised values show that APEIs contribute ~5% of AMI settlers under baseline conditions and ~19% under the mining disturbance scenario, making the relative contribution of APEIs clear at the regional planning scale at which the paper's conclusions are drawn, independently of absolute particle counts. We acknowledge that these are aggregate metrics across all AMIs rather than per-destination-cell proportional values but consider them sufficient at the regional planning scale at which the paper's conclusions are drawn. We would also note that the ≥1,000-particle threshold serves a second and distinct role as the reciprocal exchange criterion in the network optimisation algorithm, where it defines binary connected/not-connected links for the graph structure. Proportional metrics cannot substitute for this function, as the graph algorithm requires a discrete connectivity decision; once a link clears the threshold, the full continuous particle flux value drives corridor selection via flux-weighted shortest-path routing.

On the top 10% threshold: the Figure 4 legend already states explicitly that "Black-outlined cells represent the top 10% of scenario-stable scores, shown for visualisation purposes only; this threshold does not enter the analysis, optimisation, or recommendations." The 77% figure reported in the Results is derived from this threshold, but the qualitative conclusion that high-connectivity cells are predominantly located outside existing APEIs is robust to the exact threshold chosen and does not affect any downstream analysis or recommendations.

Comment: The clustering and community-detection analyses need clearer methodological support. The manuscript should explain more directly what features of the hydrodynamic field generate the observed partitions despite uniform particle release, whether these partitions align with known CCZ biogeographic provinces, and how robust boundary assignments are to changes in dispersal kernels. Cells near community edges are unlikely to have equally certain cluster membership, and this uncertainty is likely central to the disturbance scenario.

Response: We address each of these three points in turn.

On the hydrodynamic features generating the observed partitions: we would note that identifying the specific oceanographic mechanisms responsible for the community structure is outside the scope of this paper, which characterises connectivity structure as a basis for conservation planning rather than as an investigation of the physical drivers of that structure. The community partitions are presented as emergent outputs of the dispersal model under the four simulated circulation states, aggregated to identify broad-scale structure that persists across conditions. Characterising the oceanographic barriers responsible for the partitions would require dedicated process-based hydrodynamic analysis beyond the scope of a species-agnostic regional connectivity assessment.

On alignment with known CCZ biogeographic provinces: this was already addressed in the submitted manuscript. The Discussion explicitly notes that the four-community structure "is consistent with emerging evidence that the region comprises distinct biogeographic regions rather than being a single homogeneous system" (Simon-Lledó et al. 2023, 2025) and that "population genetic structure in CCZ fauna further supports regional-scale subdivision"

(Taboada et al. 2018), while acknowledging that genetic clusters do not map directly onto the four modelled communities "as expected given that genetic structure integrates species-specific biology and historical processes over longer timescales than circulation-driven dispersal potential."

On robustness of boundary assignments to changes in dispersal kernels: community detection was performed on the connectivity network aggregated across all three simulated PLDs rather than on individual dispersal kernels. A sentence has been added to the Methods making this explicit: the resulting community structure therefore reflects dispersal patterns integrated across the full modelled range of larval durations, providing a more stable basis for community identification than PLD-specific networks would. The Methods further state that "community partitions derived from this approach are conditional on the algorithm used and should be interpreted as a comparative framework for identifying broad-scale modular structure rather than definitive or algorithm-independent ecological divisions." On per-cell boundary uncertainty specifically: the transitional zone analysis explicitly identifies cells that shift community assignment under the mining disturbance scenario, providing a direct assessment of which cells have uncertain or flexible community membership. While this is driven by source removal rather than kernel change, it directly identifies the boundary cells Reviewer 4 identifies as having uncertain membership and incorporates this uncertainty into the ICV scoring via the transitional zone indicator variable.

Comment: The disturbance scenario likely remains conservative because it treats mining effects primarily as source removal. Sediment plumes extending beyond AMIs could increase mortality for near-bed larvae and further reduce both local replenishment and regional stepping-stone exchange. This possibility needs clearer acknowledgement when discussing resilience and recovery potential. The manuscript is promising but the current methodological framework is too narrowly parameterised to fully support the strength of the headline conclusions. Broader biologically realistic sensitivity analyses are needed before the claims regarding CCZ protected-area network coherence can be considered robust.

Response: On sediment plumes: the submitted manuscript already acknowledged that "the mining scenario is source-loss only and does not capture sediment plumes or acoustic disturbance beyond contract boundaries; where such impacts do extend further, wider buffers may be warranted, though empirical constraints on plume extent in the CCZ are currently insufficient to prescribe specific dimensions." We agree with Reviewer 4 that the potential for sediment plumes to increase near-bed larval mortality, and thereby further reduce regional stepping-stone exchange beyond what source removal alone captures, warrants more explicit acknowledgement. We have therefore added text to this sentence to note that plume-driven larval mortality represents an additional mechanism by which the disturbance scenario may understate connectivity impacts, and that the source-removal scenario should therefore be interpreted as a conservative lower bound on mining-related connectivity disruption rather than a complete representation of disturbance effects.

On the broader concern that the methodological framework is too narrowly parameterised to support the headline conclusions: as noted above, we have made extensive revisions to the PLD justification and vertical depth assumption, including new empirical analysis, and have been explicit throughout that conclusions are conditional on the modelled framework. We would also reiterate that the manuscript's claims have been substantially moderated since the previous round of review: results are framed as hypotheses based on modelling outputs, the title has been revised from 'reveals' to 'suggests', and the paper is explicit that it addresses

structural dispersal pathways rather than demographic sufficiency. We consider the strength of the claims made to be appropriate and proportionate to the modelling framework employed, and hope that the extensive revisions made in this round adequately address Reviewer 4's concerns.

Comment (p4, Results opening): The 'everything is everywhere' / uniform release assumption needs stronger justification given empirical evidence that abyssal larval assemblages are spatially patchy (e.g., Kersten et al. 2019).

Response: The rationale for the uniform release assumption was already present in the submitted manuscript. The Introduction states that this approach "reveals all physically plausible dispersal pathways without embedding uncertain ecological assumptions (e.g. spatially variable larval production; habitat suitability)", spatial patchiness in larval production being precisely the kind of uncertain ecological assumption the method is designed to avoid imposing in data-limited systems where habitat suitability and larval production are poorly constrained. The peer-reviewed methodological basis for this approach is established in James et al. (2025), which is cited in the manuscript.

We would also note that the Discussion provides a direct logical counter to the patchiness concern: "if connectivity is limited under uniform release, biological or environmental constraints are unlikely to strengthen it." Spatial heterogeneity in larval production would reduce connectivity in some areas relative to the uniform release baseline, making the core finding of limited inter-APEI exchange conservative with respect to patchiness rather than undermined by it. POC-scaled or habitat-weighted larval production could shift corridor priorities for particular taxa, and we acknowledge this explicitly, but it would not alter the fundamental structural finding that inter-APEI gaps exceed near-bed dispersal capacity.

Comment (p6, Table 1 / AMI replenishment): Replace or complement the ≥1,000 particle threshold with proportional source contribution metrics. Absolute counts alone may obscure biologically meaningful relative supply.

Response: This point is addressed in our response above to Reviewer 4's comment on connectivity quantification. In brief, the submitted manuscript already reported source-normalised and sink-normalised flux metrics alongside the presence-based threshold metric in Table 1 and Extended Data Table 1. The ≥1,000-particle threshold additionally serves a necessary analytical function as the reciprocal exchange criterion in the network optimisation algorithm, where proportional metrics cannot substitute for a discrete connectivity decision. We refer the reviewer to our fuller response above.

Comment (p8, Figure 2): Clarify whether dominance of unprotected sources is partly a geometric consequence of proximity to AMIs under limited dispersal assumptions.

Response: This was already addressed explicitly in the submitted manuscript. The Discussion states: "The dominance of unprotected areas as modelled particle sources under disturbance conditions is in part a geometric consequence of how the APEIs were originally placed: designed for habitat representativity and broad ecological resilience rather than larval replenishment of mining areas. Their positions were constrained by pre-existing exploration contracts, leaving central unprotected areas better positioned hydrodynamically to supply

AMIs under near-bed circulation." The Discussion further notes that this outcome is therefore "unsurprising" given the original placement criteria, while pointing out that many of the same central unprotected areas also disproportionately exhibit high scenario-stable connectivity scores, suggesting their conservation relevance extends beyond the geometric proximity argument alone.

Comment (p10, Figure 3): Clarify what network properties define communities and what hydrodynamic barriers create these partitions despite uniform release.

Response: This point is addressed in our response above to Reviewer 4's comment on the clustering and community-detection analyses. In brief, the network properties defining the communities - modularity, internal versus external connectivity, and the fast-greedy algorithm applied to the aggregated dispersal network - are described in the Methods. Identifying the specific hydrodynamic barriers responsible for the partitions is outside the scope of this paper, which characterises connectivity structure as a basis for conservation planning rather than as an investigation of its physical drivers. We refer the reviewer to our fuller response above.

Comment (p12, Figure 4): Justify the choice of 'top 10%' high-connectivity cells and report the fraction of prioritised area outside AMIs and APEIs more explicitly.

Response: On the top 10% threshold: this point is addressed in our response above to Reviewer 4's comment on connectivity quantification. In brief, the Figure 4 legend already states explicitly that the threshold is "shown for visualisation purposes only" and "does not enter the analysis, optimisation, or recommendations." The 77% figure is derived from this threshold but the qualitative conclusion - that high-connectivity cells are predominantly outside existing APEIs - is robust to the exact threshold chosen.

On the fraction of prioritised area outside AMIs and APEIs: we interpret "prioritised area" as referring to either the high-connectivity cells shown in Figure 4 or the corridor cells identified by the optimisation. For the former, the submitted manuscript reports that 77% of the top 10% cells fall outside existing APEIs, with many occurring along AMI margins: the threshold being illustrative rather than defining a formal prioritised set. For the corridor cells, which represent the analytically prioritised output of the study, the submitted manuscript already reported this breakdown explicitly: of the 53 corridor grid cells, 29 did not overlap AMIs, while 24 partially intersect existing contract areas; in aggregate, 11% of the total proposed corridor area overlaps AMIs. In either interpretation, the spatial relationship between prioritised areas and existing protected and mining zones is reported in the manuscript.

Comment (p16-18, Discussion constraints): Current assumptions are not yet sufficiently well supported. Explicit uncertainty analyses across PLD and vertical position would be more robust than relying on a narrow assumed range.

Response: This point is addressed in full in our response above to Reviewer 4's principal concern regarding the parameter space and uncertainty treatment. In brief, we have substantially expanded the PLD justification in the Methods, grounding the 69-day ceiling in the Hilário et al. (2015) cross-taxon synthesis and its specific recommendation for MPA planning, and have added new empirical HYCOM velocity profile analysis, larval swimming

speed reachability analysis, and flow direction analysis to provide an empirical rather than solely assertive basis for the near-bed confinement assumption. The Discussion constraints section has also been revised to be consistent with this more nuanced framing, explicitly stating that results are conditional on the modelled framework and that the near-bed depth constraint should not be interpreted as an upper bound on dispersal potential for all CCZ taxa. We refer the reviewer to our fuller response above.

Comment (p18, Discussion): Acknowledge that short PLDs and strict BBL confinement may systematically understate connectivity for taxa with longer-duration or off-bottom larvae.

Response: This acknowledgement is now present in the revised Discussion. The revised vertical behaviour paragraph explicitly states that reported dispersal distances "should be interpreted as conditional on the near-bed depth constraint rather than as upper bounds on dispersal potential for all CCZ taxa," and that taxa with sustained upward swimming capacity and longer PLDs could access faster currents in different directions, representing dispersal pathways not captured by this framework. The revised text therefore directly acknowledges that the modelled framework may understate connectivity for taxa with longer PLDs or off-bottom larval behaviour, while explaining why empirical constraints on these taxa are currently insufficient to parameterise these strategies within a species-agnostic framework. We refer the reviewer to our response to the principal concern above for the full details of these revisions.

Comment (p20-22, Methods): The rationale for using only January and July in two years needs evidence that these months are representative of broader circulation states.

Response: The rationale for the simulation periods was already provided in the submitted manuscript. The particle tracker Methods section states that simulations were run for January and July in 2019 and 2023 "to capture inter- and intra-annual variability, including El Niño/La Niña phases" (Extended Data Table 2). Extended Data Table 2 documents the ENSO phase classification for each simulation period using NOAA Oceanic Niño Index values, with four NOAA references: January 2019 (weak El Niño developing), July 2019 (weak El Niño transitioning to neutral), January 2023 (weakening La Niña), and July 2023 (El Niño developing). The four periods were therefore selected to contrast both seasonal extremes and contrasting ENSO states rather than sampling a single circulation regime, and results are aggregated across all four periods to reduce sensitivity to any single state. The Methods now additionally acknowledge explicitly that this sampling does not encompass the full range of ENSO variability nor longer-term decadal circulation patterns, and that results should be interpreted as conditional on the circulation states sampled rather than as long-term climatological averages: a caveat also added to the Discussion at Reviewer 1's suggestion.

Comment (p21-22, Vertical depth assumption): The argument for constraining particles to bottom flow is not convincing for all taxa. Explore shallower larval depths as a formal sensitivity analysis.

Response: This point is addressed in full in our response above to Reviewer 4's principal concern. In brief, we have added new empirical HYCOM velocity profile analysis, larval swimming speed reachability analysis, and flow direction analysis to provide an empirical

rather than solely assertive basis for the near-bed confinement assumption. We would also reiterate the practical constraint on formal sensitivity testing at shallower depths: HYCOM velocity outputs at abyssal depths span 1,000 m vertical intervals, meaning off-bottom transport cannot be parameterised at ecologically meaningful depth resolution even if sufficient biological data existed to justify it. Beyond this, parameterising vertical behaviour would require assumptions about swimming speed, duty cycle, physiological tolerances, and depth-dependent behaviour - none of which are empirically constrained for CCZ nodule fauna - and introducing these unconstrained parameters risks compounding rather than reducing model uncertainty. We refer the reviewer to our fuller response above.

Comment (p25-26, Accounting for variation / PLD): Quantify how the connectivity matrix changes as a function of PLD rather than only changes in dispersal-distance kernels.

Response: We respectfully suggest that this comment reflects a different framing of the paper's objectives than intended. This study is explicitly designed as a species-agnostic regional planning framework: one that characterises the structural connectivity landscape of the CCZ across a biologically grounded PLD range, rather than predicting connectivity for any specific taxon. In this context, the relevant question is not how the connectivity matrix varies between individual PLDs, but whether the structural conclusions about the APEI network are robust across the modelled range as a whole. Extended Data Figure 1 and Table 1 demonstrate that they are: median dispersal distances remain below 100 km across all three PLDs, the pattern of limited inter-APEI exchange holds throughout, and the dominance of unprotected non-mining areas as larval sources shows only quantitative rather than qualitative variation across PLDs. The framework aggregates across PLDs precisely to avoid producing outputs contingent on any single taxon's dispersal duration. This is a deliberate design choice for a planning tool intended to be applicable across the taxonomic diversity of CCZ nodule fauna, for whom empirical PLD data are absent. Per-PLD connectivity matrix comparisons would be more appropriate in a species-specific modelling exercise, which is exactly the kind of targeted analysis the framework is designed to support and enable as species-specific empirical data accumulate. The peer-reviewed methodological basis for this species-agnostic approach is established in James et al. (2025), cited in the manuscript.

Comment (p27-28, Community detection): Provide robustness checks or confidence measures for community assignment, especially for boundary cells where membership may be uncertain.

Response: This point is addressed in our response above to Reviewer 4's comment on the clustering and community-detection analyses. In brief, three things already in the submitted manuscript speak to this concern. First, community detection was performed on the connectivity network aggregated across all three simulated PLDs rather than on individual dispersal kernels. A sentence has been added to the Methods making explicit that the resulting community structure therefore integrates across the full modelled range of dispersal capacity, providing a more stable basis for community identification than PLD-specific networks would. Second, the Methods explicitly state that "community partitions derived from this approach are conditional on the algorithm used and should be interpreted as a comparative framework for identifying broad-scale modular structure rather than definitive or algorithm-independent ecological divisions." Third, and most directly relevant to boundary cell uncertainty, the transitional zone analysis explicitly identifies cells that shift community assignment under the mining disturbance scenario. While this is driven by source removal

rather than by changes in dispersal kernels, it directly identifies and quantifies the boundary cells with uncertain or flexible community membership - precisely the cells Reviewer 4 identifies as problematic - and incorporates this uncertainty explicitly into the ICV scoring via the transitional zone indicator variable, meaning boundary uncertainty is not ignored but actively used to inform corridor prioritisation.

Comment (p29, Mining scenario): Expand disturbance scenarios to discuss potential mortality effects of sediment plumes on near-bed larvae, not only source removal. This possibility needs clearer acknowledgement when discussing resilience and recovery potential.

Response: We agree and have expanded the Discussion accordingly. The submitted manuscript already acknowledged that the mining scenario is source-loss only and does not capture sediment plumes or acoustic disturbance beyond contract boundaries. We have added text to explicitly acknowledge that sediment plumes could increase near-bed larval mortality beyond contract boundaries, representing an additional mechanism by which the disturbance scenario may understate connectivity impacts, and that the source-removal scenario should therefore be interpreted as a conservative lower bound on mining-related connectivity disruption rather than a complete representation of disturbance effects.
